# A High‐Throughput Live Imaging Platform to Investigate Circuit‐Dependent Regulation of Circadian Rhythms in Brain Tissue

**DOI:** 10.1002/advs.75427

**Published:** 2026-04-28

**Authors:** Marco Ferrari, Natalie Ness, Julieta Acosta, Marco Brancaccio

**Affiliations:** ^1^ UK Dementia Research Institute at Imperial College London London United Kingdom; ^2^ Department of Brain Science Imperial College London London United Kingdom

**Keywords:** brain tissue, circadian rhythms, live‐imaging

## Abstract

Circadian function in multicellular organisms arises from coordinated interactions amongst diverse cellular tissue populations. Existing approaches for long‐term imaging of within‐tissue circadian regulation remain low‐throughput, highly specialized, and largely inaccessible. Here, we developed ClockCyte, a high‐content fluorescent live‐imaging platform that enables continuous monitoring of circadian rhythms in up to 144 brain tissue samples. Using the mouse suprachiasmatic nucleus as a model, ClockCyte captures the differential circadian tissue regulation of neurons and astrocytes. We further identified a previously uncharacterized oscillatory circadian compartment in axonal calcium, showing highly homogeneous activity, opposed to waves of intracellular neuronal calcium. By deleting Bmal1 in neurons, we reveal the network underpinnings connecting clock gene expression to network‐wide axonal regulation. The discovery of distinct circadian properties of axonal calcium and their disruption by Bmal1 ablation highlights the potential to reveal new principles of intra‐tissue network‐level circadian organization. More broadly, this approach will enable systematic explorations of how cell‐type‐specific and compartmentalized subcellular rhythms contribute to brain physiology.

## Graphical abstract

Biological rhythms coordinate physiology, from genes to behavior. Study of circadian rhythms in brain tissue is constrained by limited throughput and spatial and temporal information quality. A new platform for high‐throughput, long‐term multiplexed fluorescent live imaging of circadian rhythms in brain slices is introduced. Using this platform, specific circadian rhythmic features of calcium in axons, as opposed to overall intracellular neuronal calcium, are revealed.

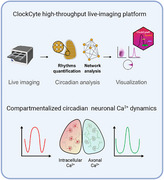

## 1 Introduction

Circadian clocks are cell‐autonomous oscillators that orchestrate daily rhythms in gene expression, physiology, and behavior. In multicellular organisms, these rhythms emerge from complex, distributed networks of interacting cells and tissues [1]. While the importance of circadian clocks in key aspects of tissue homeostasis and dyshomeostasis in disease is increasingly recognized [2, 3, 4, 5, 6], much of our current mechanistic understanding derives from low‐throughput bulk‐measurement paradigms, better suited to characterize population‐level behavior rather than within‐tissue circadian regulation. Existing technologies for monitoring circadian oscillations in explanted brain tissue, such as photomultiplier tube (PMT)‐based luminometry, offer high sensitivity but are fundamentally limited by the lack of spatial information, single‐sample capacity, and ultimately the inability to assess reciprocal interactions between different resident cells within tissues [7].

Very few imaging systems exist that have the necessary sensitivity and stability to continuously monitor circadian rhythms in tissues over prolonged timespans, and are achieved by highly specialized custom‐made setups [8, 9], or technically complex microscopes (e.g., Olympus/Evident LV200, now discontinued) [10, 11], only available in a few dedicated chronobiology laboratories worldwide. Thus, investigations in tissue regulation of circadian function are generally fraught with very low experimental throughput, considerable costs (both referred to the specialized equipment and to the time charges for long‐term usage of shared imaging facilities), as well as high know‐how barriers. These limitations restrict the investigation of tissue‐level circadian rhythmicity to a handful of specialized circadian laboratories, de facto creating a major logistical barrier to investigations of circadian medicine and to exploring the targetability of circadian tissue homeostasis for health and longevity [12, 13].

To address this gap, we have developed ClockCyte, a novel high‐throughput live‐imaging platform enabling continuous, long‐term monitoring of circadian rhythms in up to 144 tissue explants over several weeks in multiple fluorescent channels. ClockCyte is based on a widely available commercial live‐cell imaging system, targeted to cell culture applications. We validated this system for long‐term multiplexed continuous recording of circadian oscillations within brain tissue. We designed open‐source R‐based analytical pipelines to enable the rapid extraction of basic circadian parameters using a user‐friendly interface, as well as specialized pipelines to investigate complex spatio‐temporal features emerging from circadian network regulation across the tissue. As a testbed for validating the ClockCyte platform, we used organotypic cultures of the hypothalamic suprachiasmatic nucleus (SCN). The SCN is the master circadian clock in mammals, playing a key role in internally synchronizing circadian physiology and aligning it to the light‐dark cycle [14]. Daily orchestration of physiology and behavior in mammals ultimately depends on the robust circadian output generated by the SCN circuit, emerging from the specific contributions of the different neuronal and astrocytic subpopulations present within the tissue [3, 4, 15, 16, 17]. Remarkably, the SCN can retain circadian cycles of clock gene expression, cell‐type‐specific activities (e.g., neuronal, astrocytic), and neurotransmitter release when isolated in tissue culture [3, 4, 10, 11, 15].

We used the ClockCyte to monitor circadian rhythms with established genetically encoded reporters of astrocytic and neuronal activity [10], reproducing previously reported neuronal‐astrocytic anti‐phasic oscillations [4, 15]. Moreover, by implementing new reporters of axonal calcium, we demonstrated an unforeseen subcellular compartmentalization of calcium signals across the SCN circuit. Finally, we investigated how ablation of the essential clock gene Bmal1 in SCN neurons affects the spatiotemporal network dynamics of SCN axonal oscillations and found an unexpected early role of Bmal1 in disrupting long‐range axonal connectivity within the tissue, with cellular and local rhythmicity only affected at later stages.

## 2 Results

### 2.1 Establishment of Long‐Term Live Fluorescent Imaging of Circadian Rhythms in SCN Tissue

**FIGURE 1:**
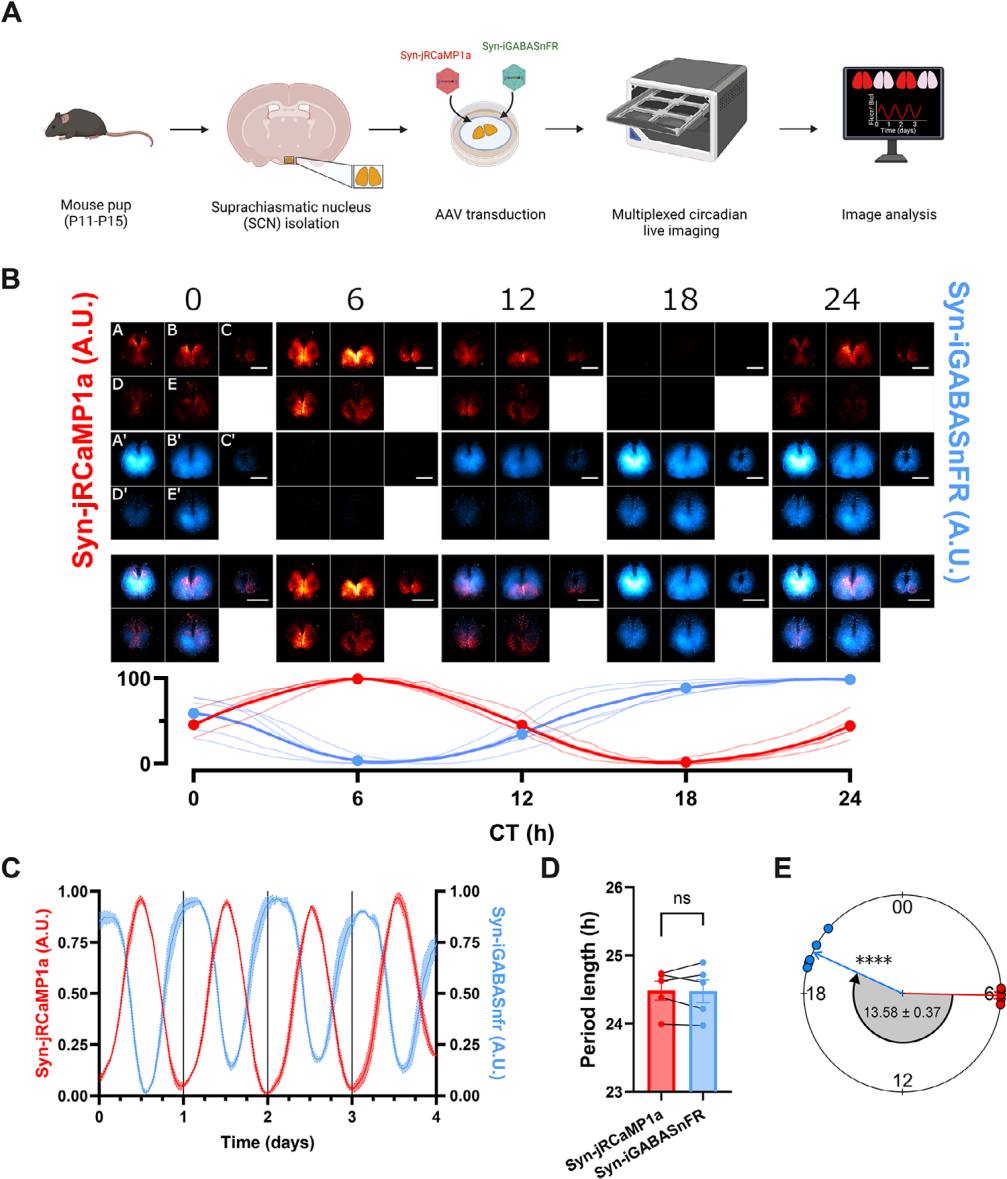
Multiplexed imaging of circadian function in SCN tissue in the Incucyte system. (A) Schematic of the experimental workflow. (B) Montage of 5 co‐recorded SCN slices, simultaneously expressing a genetically encoded neuronal Ca^2+^ reporter (top row, Syn‐jRCaMP1a, red) and an extracellular GABA reporter (middle row, Syn‐iGABASnFR, cyan). Merged images are displayed in the bottom row. Individual traces and mean traces are shown in the bottom plot (normalized values). Scalebar = 500 µm. (C) Mean traces of Syn‐jRCaMP1a and Syn‐iGABASnFR monitored over several days. Normalized values, n = 5. (D) Period length of Syn‐jRCaMP1a and Syn‐iGABASnFR circadian oscillations detected with the FFT‐NLLS method. Two‐tailed paired *t*‐test. ns, *p* >0.05. (E) Rayleigh plot of Syn‐jRCaMP1a and Syn‐iGABASnFR phases. Two‐tailed paired t‐test. ^****^ = *p* < 0.001.

We isolated SCN brain slices from P11‐P15 mouse pups and transduced them sequentially with AAVs expressing GABA reporter (pAAV‐hSyn‐iGABASnFR) and a calcium reporter (pAAV‐hSyn‐jRCaMP1a), both expressed in neurons by the hSyn promoter. Slices were cultured in 6‐well plates and imaged continuously every 30 min with signals in the green, red, and phase channels to validate the setup capability of simultaneously detecting circadian oscillations of intracellular neuronal calcium and extracellular GABA (Figure 1A; Movie S1). All SCN samples produced strongly rhythmic circadian oscillations in both reporters, which could be continuously monitored over several days (Figure 1B,C). Neuronal Ca^2+^ and extracellular GABA had a period of ∼24.5 h (Figure 1D), which was consistent across co‐expressed reporters. On the other hand, neuronal and GABA rhythms had distinct waveforms and contrasting phases, respectively peaking at circadian times (CT) CT6 and CT19, as expected (Figure 1C,E) [4, 18]. Thus, our implementation of continuous tissue‐level imaging returned stable fluorescent signals (Figure S1A,B) and was able to capture circadian oscillations of different mediators and neurotransmitters within the same SCN over several weeks, with no appreciable spillover/ interference across different fluorescent channels (Movie S1).

### 2.2 Enabling High‐Throughput Detection and Analysis of Circadian Rhythms in SCN Tissues

**FIGURE 2:**
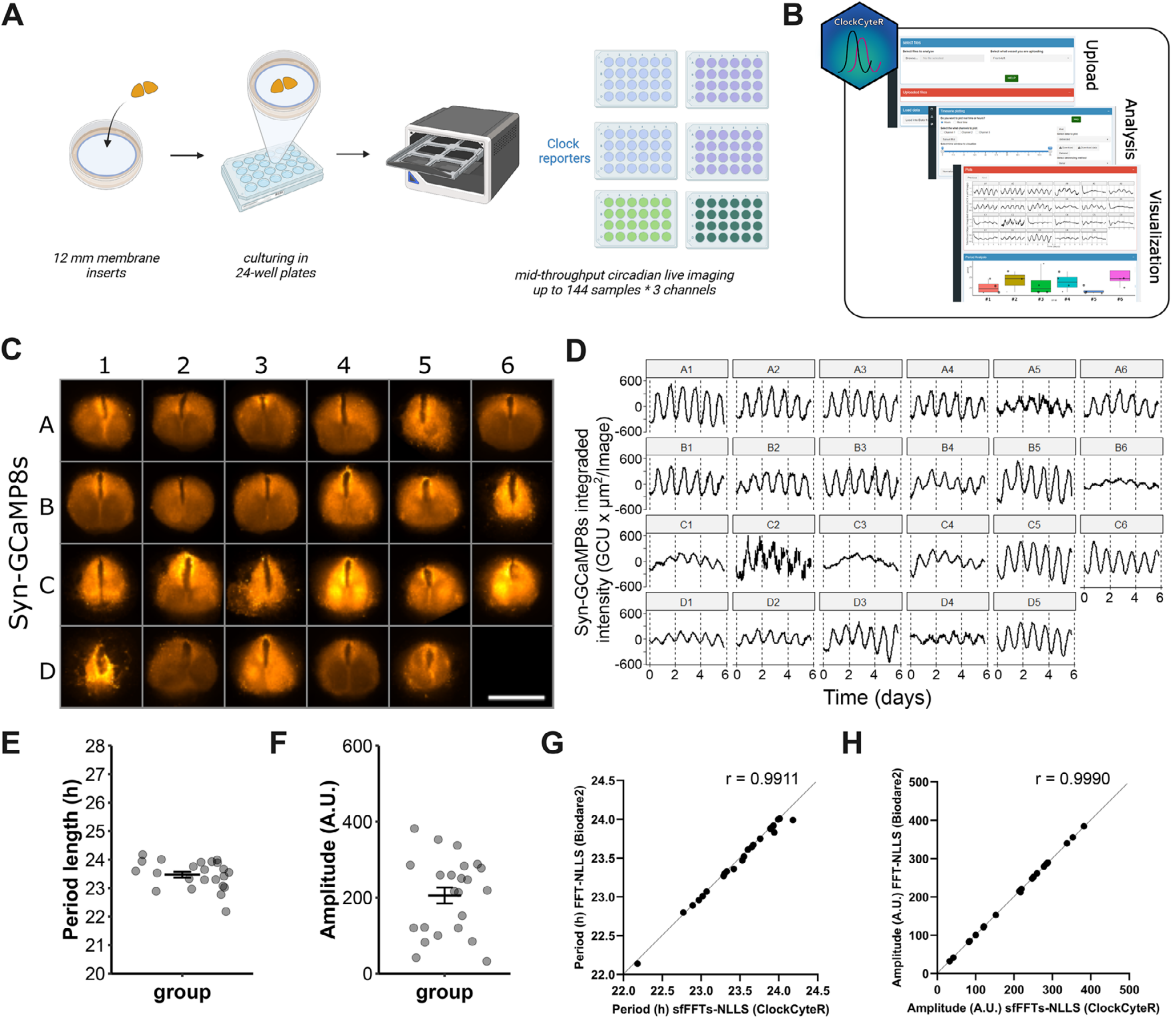
Establishing a new imaging platform for high‐content quantification of circadian rhythms in SCN tissue. (A) ClockCyte workflow: isolation and culturing of brain slices on membranes for 24‐well plates, simultaneous recording of multiple plates, and circadian analysis using the (B) *ClockCyteR* package. (C) Micrographs of SCN organotypic slices transduced with Syn‐jGCaMP8s, reporting neuronal Ca^2+^, were simultaneously recorded for several days in 24‐well plates. Orange, scalebar = 1 mm. (D) Syn‐jGCaMP8s traces plotted using *ClockCyteR* (linear detrending applied). (E,F) Period length and amplitude quantification of Syn‐jGCaMP8s using the sfFFTs‐NLLS algorithm. Mean±SEM, n = 23. (G) Spearman correlation of period length quantification using FFT‐NLLS algorithm from Biodare2 (*y*‐axis) and sfFFTs‐NLLS ClockCyteR (*x*‐axis), r = 0.9911. (H) Correlation of amplitude quantification using FFT‐NLLS algorithm from Biodare2 (*y*‐axis) and sfFFTs‐NLLS ClockCyteR (*x*‐axis), r = 0.9990.

Experimental capacity is a major bottleneck in circadian experiments, with most lasting several weeks, not compatible with shared facility usage. To address this limitation, we implemented an improved organotypic culture system by using 24‐well plates, which enabled the simultaneous recording of up to 144 independent tissue samples in three different channels within a single experimental session (Figure 2A; Movie S2). A second significant barrier is the multivariate analysis of such a high number of rhythmic time series, especially for non‐specialized laboratories. To tackle this, we developed an interactive app based on the R Shiny framework [19] for the analysis of time series (Figure S2A). *ClockcyteR* allows for fast and semi‐automated data pre‐processing, analysis, and plotting (Figure 2B). Following file upload (Figure S2B), time series can be pre‐processed, removing possible outliers, detrending, smoothing, normalizing the data, and restricting the time interval considered (Figure 2B; Figure S2C). Each transformation step can be plotted, and the period analysis can be applied to either raw traces or transformed data. We used a dataset obtained from the recording of Ca^2+^ activity (Syn‐GCaMP8s) in 23 SCN slices (Figure 2C) over 6 days to illustrate the platform functioning. An overview plot generated using *ClockCyteR* allows simultaneous visualization of the Ca^2+^ signal in the entire dataset (Figure 2D), and annotated plots of individual traces can be generated and exported (Figure S2D). Key circadian parameters, such as period length and amplitude, can be quantified using a single‐frequency Fast Fourier Transform‐seeded Non‐Linear Least Squares (sfFFTs‐NLLS) algorithm and plotted (Figure 2E,F, and Methods). We benchmarked our implementation of the FFT‐NLLS algorithm (sfFFTs‐NLLS) against Biodare2 [20] (Figure 2G,H) and found a very high correlation (r = 0.99) for the quantification of both circadian periodicity and amplitude. Additional standard algorithms for the quantification of circadian parameters, such as the Lomb–Scargle and Chi‐Square from the zeitgeber package [21], were added to *ClockCyteR*to perform alternative options for period analysis (Figure S2G).

### 2.3 Spatiotemporal Characterization of Intracellular vs. Axonally‐Enriched Ca^2+^ Reporters in SCN Slices

**FIGURE 3:**
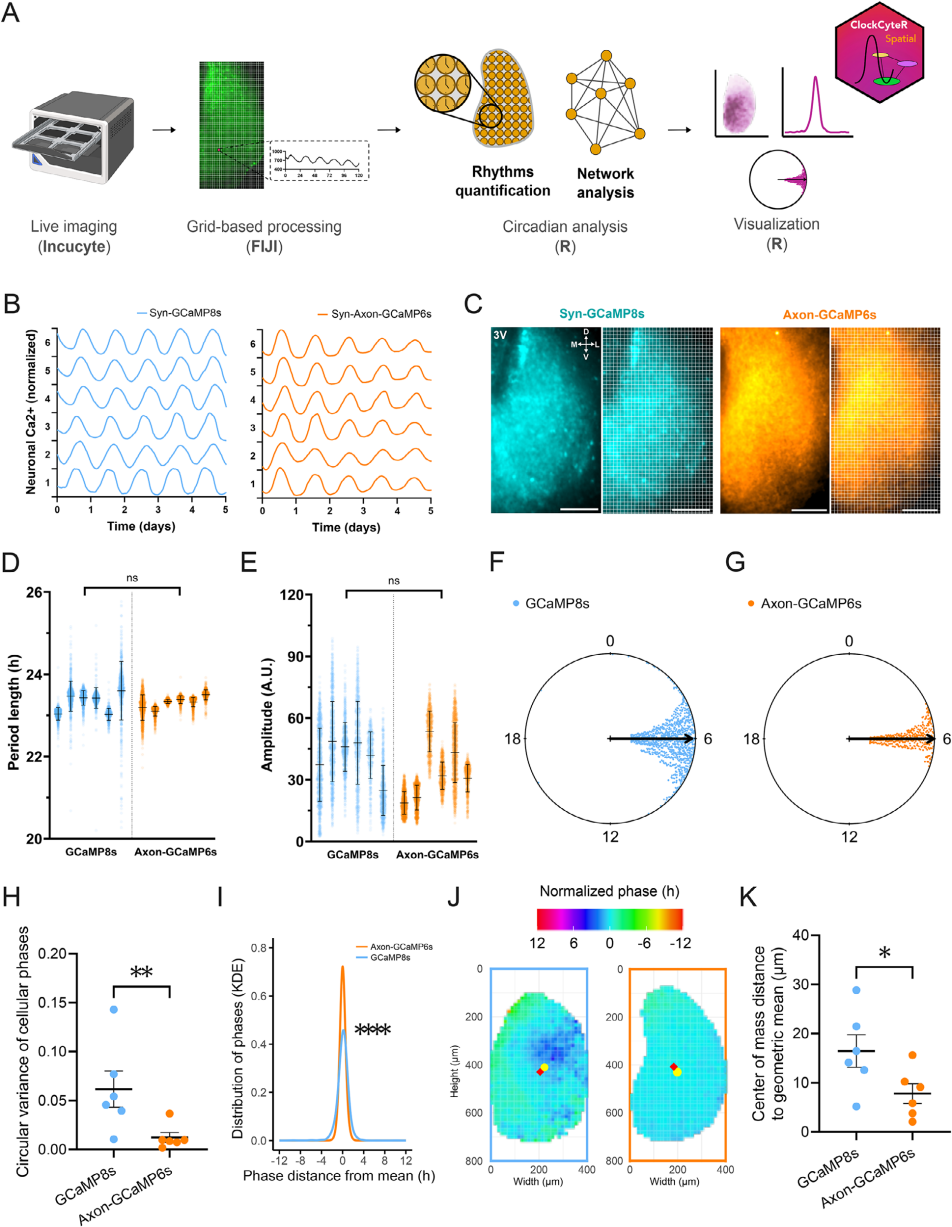
Dissection of the spatiotemporal circuit properties of intracellular vs. axonal calcium in SCN tissue. (A) Diagram of ClockCyteR.spatial pipeline workflow and output. (B) Intracellular neuronal Ca^2+^ (monitored by Syn‐GCaMP8s, left), and Axonal Ca^2+^ (Syn‐Axon‐GCaMP6s, right) traces from SCN slices. Normalized and spaced traces. (C) Representative micrographs of SCN slices transduced with AAV‐Syn‐jGCaMP8s (cyan, left), and AAV‐Syn‐Axon‐GCaMP6s (orange, right), and superimposed grids used to extract spatiotemporal information from the live imaging recordings. Scalebar = 150 µm. (D) Nested scatter plots of neuronal Ca^2+^ period, and (E) amplitude monitored in each SCN slice (mean±SD, n = 6 per group, avg. 799 ROIs per sample, two‐tailed nested t‐test). (F‐G) Representative Rayleigh plots of intracellular Ca^2+^ and axonal Ca^2+^. Each point represents one ROI. H) Circular variance of ROI phases in each sample across the two reporters (mean±SEM, Mann–Whitney test, two‐tailed). (I) Comparison of phase distribution (KDE) across the two reporters; (n = 6 per group, 4936 and 4811 ROIs, respectively. Kolmogorov–Smirnov test. (J) Spatiotemporal maps of neuronal Ca^2+^ phase in the SCN. Intracellular Ca^2+^ (cyan border, left) and axonal Ca^2+^ (orange border, right). In each map, the red and the yellow dots represent the geometric mean and the centre of mass of phase values, respectively. (K) Centre of mass distance to the geometric mean (µm) (mean±SEM, n = 6 samples per reporter, two‐tailed unpaired t‐test). ^*^ = *p* < 0.05, ^**^ = *p* < 0.01, ^****^ = *p* < 0.0001, ns = *p* >0.05.

While the 24‐well configuration coupled with ClockCyteR analysis of time series enables the rapid scoring of multiplexed circadian parameters in several samples, it only captures mean collective properties, without fully leveraging the high‐quality data‐rich single‐cell readouts embedded within the recordings of SCN tissue (Movie S1). As different cell types can express different rhythmicity in the SCN, and intercellular communications are critically important for maintaining robust circadian timekeeping in mammals [10, 15], we established a second analysis pipeline (ClockCyteR.spatial) to extract circadian parameters within the tissue and capture circuit‐level circadian behavior (Figure 3A; Figure S3A–D, and Methods). We used this pipeline to examine whether the circadian network behavior of neuronal calcium compartments may be differentially regulated in the SCN. To do so, (i) we established a new reporter to monitor circadian activity of axonal calcium and demonstrated its distinct network properties when compared to intracellular neuronal calcium; (ii) we performed an in‐depth real‐time analysis to reveal how this newly identified axonal calcium compartment is dynamically altered over time when essential clock genes are ablated in SCN neurons.

Ca^2+^ dynamics can be monitored using different genetically encoded Ca^2+^ reporters (GECI). Comparing the expression of clock gene reporter PER2::LUC [22] to different neuronal Ca^2+^ reporters (Figure S4A,B) (by using the Olympus LV200 microscope to reveal bioluminescence signals), we found no significant differences in circadian period length or relative phase across the different calcium reporters (including Axonal‐GCaMP6s [23], see below) (Figure S4C,D). By using the voltage reporter ARCLightD (Syn‐ARCLightD) [24], we monitored circadian variations of neuronal membrane potential (Figure S5A,B), which were also phase‐aligned to intracellular Ca^2+^ rhythms [15] (Figure S5C,D). Interestingly, the distribution of phases identified by ROI analysis (Figure S5E,F) highlighted a sharper distribution for Syn‐ARCLightD compared to Syn‐jRCaMP1a (Figure S5G,H). These data, acquired by ClockCyte, confirmed previous findings showing synchronous voltage rhythms in the SCN, opposed to waves of intracellular calcium rhythms, lasting several hours [25].

To reveal any compartmentalized circuit‐level regulation of neuronal calcium, we closely monitored axonal Ca^2+^ using the Syn‐Axon‐GCaMP6s (Axon‐GCaMP6s) reporter, enriched in axons [23]. Analysis of fluorescence particle size demonstrated it stained smaller signals, consistent with neurite‐sized particles, as opposed to the co‐expressed intracellular calcium reporter Syn‐jRCaMP1h, which mostly returned cell‐size areas (Figure S6A,B). In addition, Axon‐GCaMP6s fluorescence showed increased colocalization with the SCN presynaptic marker Synaptophysin 1 [26, 27, 28], supporting enrichment of the reporter within presynaptic compartments (Figure S6C,D). While analysis of mean traces (Figure 3B) did not return any significant difference in period or amplitude (Figure S7A,B); the waveform of the axonal Ca^2+^ signal appeared sharper when compared to intracellular Ca^2+^, as indicated by the smaller area under the curve (AUC) of the cycle waveform (Figure S7C,D). Using ClockCyteR.spatial to investigate whether this narrower waveform may reveal an underpinning differential regulation of compartmentalized Ca^2+^ within the SCN, we performed network‐level analysis of the two reporters. The time‐lapse recordings were parsed through a grid (Figure 3B), and the corresponding signal from each ROI (approximating one cell) was extracted into a time series array. The features of Ca^2+^ rhythms from every ROI are shown in nested plots (period length, amplitude, Figure 3D,E). We observed no significant differences in period length between the two reporters. However, the distribution of cellular circadian phases was different between the two reporters: circular statistical analysis using a Rayleigh test showed reduced circular variance of axonal calcium when compared to intracellular Ca^2+^, with the intracellular reporter having a significantly larger variance of phase values (Figure 3F–H). The cellular phases from the axonal Ca^2+^ reporter were more tightly clustered around the mean phase, as revealed by the sharper peak of the kernel density estimate (KDE, Figure 3I). To reveal whether these differential phase distributions of axonal vs. intracellular calcium may be indicative of a differential circuit‐level regulation of compartmentalized calcium across the SCN, we extracted the normalized phase distribution across the SCN space. This analysis revealed that axonal Ca^2+^ has a uniform phase across the slice, compared to intracellular Ca^2+^ reporter, which is instead organized in spatiotemporal waves (Figure 3J) progressing through the tissue, reflected by a difference between the distance between the geometric center of the tissue and the center of mass of cellular phases (Figure 3K). Interestingly, while neuronal intracellular Ca^2+^ has been previously shown to be organized in spatiotemporal waves, akin to clock gene expression [4, 15], calcium in axons is shaped in a pulse‐like uniform distribution (see Discussion).

### 2.4 BMAL1 Deletion Disrupts Circadian Oscillations of Axonal Ca^2+^ in SCN Slices

**FIGURE 4:**
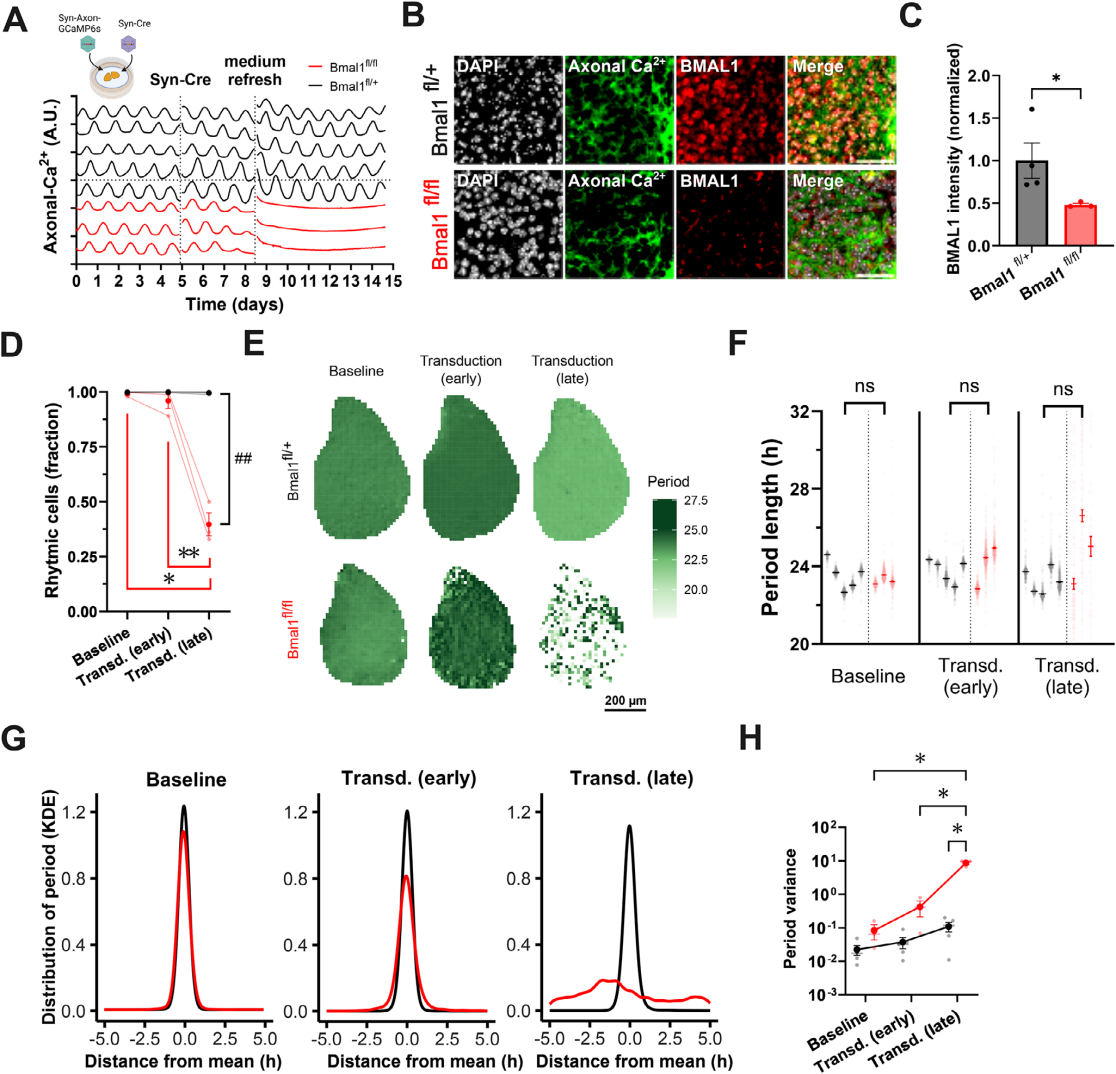
Bmal1 ablation impairs circadian oscillations of axonal calcium. (A) Normalized traces of AAV‐Syn‐Axon‐GCaMP6s expressed in SCN isolated from Bmal1^fl/+^ heterozygotes (black) and Bmal1^fl/fl^ homozygotes (red) before and after transduction with AAV Syn‐Cre, deleting the floxed Bmal1 allele. (B) Representative image of ROI in SCN slices after treatment with Syn‐Cre (Bmal1^fl/+^, top; Bmal1^fl/fl^, bottom). Labelled with DAPI and BMAL1 antisera. Scalebar = 50 µm. (C) Quantification of BMAL1 staining intensity after viral transduction (Mean±SEM, Mann‐Whitney test, one‐tailed). (D) Fraction of ROIs with a detectable rhythm (sfFFTs‐NLLS algorithm; Individual datapoints and mean±SEM; Two‐way ANOVA with Tukey multiple comparison test, F(1, 6)). (E) Spatial map of period length and normalized phase of axonal Ca^2+^ rhythms across genotypes and experimental phases; one representative sample per genotype, shown longitudinally in time. (F) Nested plot of period length of axonal Ca^2+^ rhythms across genotype and time; nested one‐way ANOVA. (G) Comparison of period distribution (KDE) across the two genotypes at baseline (left, Wasserstein distance effect size: 0.11 h), early transduction (middle, effect size:0.31 h), and late transduction (right, effect size: 3.77 h). (H) Period variance of axonal Ca^2+^ rhythms; Two‐way ANOVA with Tukey multiple comparison test, F(1, 6). In panels A, C, D, F‐H, n = 3 for Bmal1^fl/fl^ and n = 5 for Bmal1^fl/+^ respectively. ^*^ = *p* < 0.05, ^**^ = *p* < 0.01, ## = *p* < 0.01, ns = *p* >0.05.

To functionally investigate the effects of genetic clock manipulation on circuit‐level regulation of axonal Ca^2+^ rhythms in SCN slices, we isolated samples from homozygote or heterozygote floxed Bmal1 mice (Bmal1^fl/fl^ and Bmal1^fl/+^, respectively) and transduced them with the Syn‐Axon‐GCaMP6s reporter. Axonal Ca^2+^ rhythms were monitored for 5 days (baseline), after which all the samples received a neuronal‐restricted Cre recombinase (AAV‐Syn‐Cre) to respectively achieve a full or partial Bmal1 deletion [11] in neurons (Figure 4A). Bmal1^fl/fl^ samples in which the Cre recombinase excised both Bmal1 alleles became progressively arrhythmic, whereas Bmal1^fl/+^ heterozygotes retained stable circadian oscillations of axonal Ca^2+^ over the experimental timeframe, as expected (Figure 4A; Figure S8A). Immunohistofluorescence stainings conducted in SCN slices at the end of the experiment confirmed the effective Bmal1 ablation in Bmal1^fl/fl^ compared to Bmal1^fl/+^ SCNs (Figure 4B,C). To investigate how Bmal1 ablation affected circadian oscillations of axonal Ca^2+^ over time, we defined three experimental phases: (i) baseline (days 0–5), (ii) early transduction (days 5–8.5) and (iii) late transduction (days 8.5–14.5), with the early transduction phase capturing more closely the dynamic changes to circuit‐level organization of circadian SCN timing upon Bmal1 ablation. We used ClockCyteR.spatial to extract axonal Ca^2+^ oscillations, as already shown (Figure 3). The number of rhythmic axonal Ca^2+^ ROIs steadily decreased over time in Bmal1 homozygotes, but not in heterozygote SCNs, with the number of rhythmic ROIs more than halved in the late stages (Figure 4D). The mean period of the remaining oscillators was, however, not altered even at late stages (Figure 4E,F). In contrast, the period distribution of axonal Ca^2+^ (measured by KDE) became progressively shallower, and period variance significantly increased over time (Figure 4G,H). Variance of ROI phases was similarly increased in Bmal1 homozygote SCNs (Figure S8B,C).

### 2.5 BMAL1 Deletion Impairs Intercluster Network Organization and Period Coherence of Axonal Ca^2+^ in the SCN

**FIGURE 5:**
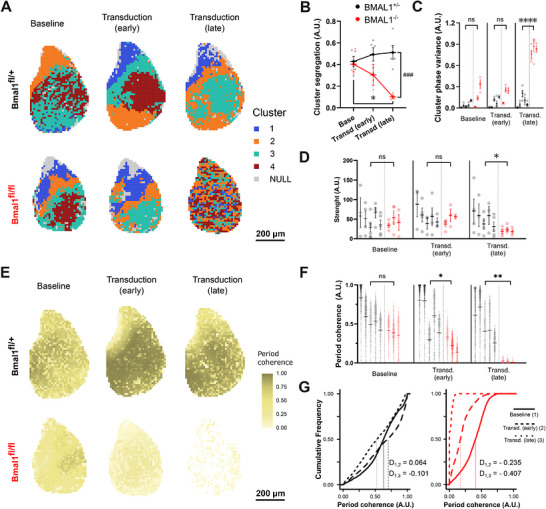
BMAL1 deletion impairs the cluster structure period coherence of the SCN circuit. (A) Spatial distribution of clusters (clusters are ordered by mean cluster phase, representative samples). (B) Quantification of cluster segregation across genotype and time. Two‐way ANOVA with Šídák's multiple comparison test, F(1, 6). (C) Nested plot of intra‐cluster phase variance (mean±SEM). Nested one‐way ANOVA with Šídák's multiple comparison test, F(5,18) = 40.35, *p* < 0.0001. (D) Nested plot of normalized cluster strength across genotype and time (mean ± SEM). Nested one‐way ANOVA F(5,18) = 1.788, p = 0.17, Fisher's LSD test. (E) Longitudinal spatial analysis of axonal Ca^2+^ period coherence; left to right: Baseline, early transduction (day 5.5–8.5), and late transduction (day 11.5–14.5). Bmal1^fl/+^ (top), Bmal1^fl/fl^ (bottom). (F) Period coherence across genotype and time. Nested One‐way ANOVA, F(5,18) = 6.58, *p* < 0.01. (G) Cumulative distribution of period coherence during baseline (continuous line), early (dashed line), and late transduction (dotted line) across genotype. n = 5, 3 for Bmal^fl/+^ and Bmal1^fl/fl^, respectively. ^*^ = *p* < 0.05, ^**^ = *p* < 0.01, ### = *p* < 0.001, ^****^ = *p* < 0.0001, ns = *p* >0.05.

To further investigate which axonal Ca^2+^ circuit properties are affected first by the deletion of Bmal1, we set up two advanced analysis pipelines to characterize dynamic changes of network function triggered by the deletion of BMAL1 in neurons: (1) local cluster structure and (2) period coherence of the SCN tissue (see Methods). Using an unsupervised clustering strategy correlating the ranks and oscillatory properties of axonal Ca^2+^ traces in space and time, we revealed that, notwithstanding the homogenous phase distribution (Figure 4), the SCN axonal signal is organized into discrete areas of increased local network coherence (“clusters”) (Figure 5A). Plotting the values of period length and normalized phase detected in each group illustrates the separation between neighboring clusters (Figure S9A,B). The spatial segregation across such clusters is a geometrical measure of how well separated these cluster areas of local coherence remain over time. Importantly, BMAL1 ablation triggered an early and progressive reduction in spatial segregation, driven by the intermingling of the clusters (Figure 5A,B). This diminished spatial organization across clusters preceded the impairment of the within‐cluster coherence, as evidenced by an increased mean cluster phase variance and diminished cluster strength (reduced total correlation of signals) within each cluster at late stages (Figure 5C,D). These results revealed that following BMAL1 ablation, axon‐mediated connectivity of more distal parts of the SCN was affected before areas of local connectivity were impacted.

Finally, in order to understand whether an alteration of the periodic organization of axonal Ca^2+^ may underpin the network impairment observed in Bmal1‐deleted SCN tissues, we monitored period coherence (defined as local similarity of an ROI's period to its neighbors) over time and found it was significantly reduced already in the early stages of Bmal1 ablation, and progressively worsened over time (Figure 5E,F). This was also confirmed by the early changes in the cumulative frequency distributions over time in Bmal1‐ablated slices compared to heterozygote controls (Figure 5G).

## 3 Discussion

In this study, we developed ClockCyte, a live‐imaging platform that enables multiplexed, long‐term monitoring of circadian oscillations within brain tissue, offering high throughput, wider accessibility, and analytical depth. Using SCN explants as a testbed, we demonstrate that the system reliably detects circadian oscillations in neuronal calcium and astrocyte‐derived GABA, reproducing known antiphase relationships which are critically important to maintain tissue‐level organization of circadian function within the SCN and stable patterns of sleep and wake in mammals [3, 4, 15, 18].

We then used the newly established imaging platform and experimental pipelines to evaluate compartmentalized neuronal calcium signals, which revealed previously uncharacterized circadian rhythms in axonal calcium: these were sharply synchronized and spatially homogeneous, as opposed to the waxing and waning of intracellular calcium rhythms, suggesting an unforeseen complexity of compartmentalized calcium circadian regulation across the SCN tissue. Finally, by combining this imaging platform with targeted genetic perturbations, we showed that neuronal Bmal1 deletion disrupts the network coherence of axonal calcium oscillations, highlighting the central role of clock gene integrity in maintaining SCN circuit stability via regulation of axonal function.

### 3.1 The Tissue is the Issue

In multicellular organisms, physiological functions emerge from tissue‐level organizations, and are deeply rooted in the finely orchestrated relations between tissue‐specific, stromal, and immune cell types embedded within a tightly regulated interstitial space [29]. Within‐tissue interactions have been recently highlighted as a cornerstone of circadian homeostasis, a key element of resilience against chronic and multifactorial diseases, ranging from metabolic disease to cancer and neurodegeneration [2, 5, 12, 30, 31, 32]. Organotypic cultures of SCN tissue are amongst the most versatile and powerful models used to investigate circadian tissue regulation. They have been critically important to determine the role of different cell types (e.g. astrocytes, neuropeptidergic subpopulations) in circadian tissue regulation, thanks to their ability to retain long‐term daily patterns of clock gene expression and functional activity, as well as their amenability to molecular/drug interventions aimed at monitoring and/or designer‐modifying circadian pathways underpinning the regulation of daily behavior [3, 4, 11, 15, 17]. While the potential of this model in neuroscience and chronobiology research is well recognized, its usage is limited to a restricted number of research laboratories, given the need of custom‐made specialized equipment and low experimental throughput, especially relevant given the length of the experiments typically spanning over several weeks, and the need for ad hoc expertise in multivariate and circular analysis of time series associated with such investigations.

### 3.2 Establishment of a New Experimental Platform to Investigate Circadian Clocks in Tissues

The *ClockCyte* platform substantially expands experimental capacity and accessibility in tissue‐level circadian research. Existing paradigms—such as photomultiplier tube (PMT)‐based luminometry or dedicated imaging setups (e.g., LV200 systems)—provide valuable but limited insight, typically constrained to single‐sample throughput, absence of spatial resolution, and dependence on bespoke laboratory infrastructure. Automated fluorescence live imaging systems continuously monitoring fluorescent signals in cells hosted within cell culture incubators offer great promise for circadian biology and have been previously used to estimate the phase resetting properties of different substances on circadian clocks in single cells [33]. By adapting the Incucyte for organotypic slice imaging, we introduce a platform capable of continuously monitoring up to 144 SCN samples in multiple fluorescent channels within standard incubator conditions. To achieve this, several technical improvements have been made to the recording setup, including the use of smaller 12 mm membranes, the optimization of culture medium volumes, and a humid chamber setup increasing long‐term thermal stability. This configuration allows simultaneous longitudinal imaging of multiple genotypes, treatments, and/or circadian reporters over months, thus vastly multiplying experimental throughput and statistical power. Beyond sheer capacity, *ClockCyte* advances data comparability and reproducibility of datasets. The use of an industrially standardized imaging system ensures consistent optical and environmental parameters across laboratories, thereby minimizing inter‐laboratory variability—a critical limitation of custom‐built circadian setups. The Incucyte allows for stable illumination and environmental control, thus providing long‐term signal fidelity and generating datasets that are both quantitatively reliable and directly compatible with industry workflows. This harmonization between academic and industrial practices offers a significant step toward translational circadian biology and circadian medicine, supporting systematic investigations of tissue‐level rhythmicity in health and disease.

### 3.3 ClockCyteR and ClockCyteR.spatial as Widely Accessible Experimental Analysis Pipelines

To complement this imaging framework, we developed two dedicated open‐source analysis tools, available as R packages: *ClockCyteR* and *ClockCyteR.spatial*. The *ClockCyteR* package and its associated Shiny graphical user interface (GUI) enable non‐specialist users to process large sets of time series data from the Incucyte. Implementing a single‐frequency FFT‐seeded NLLS algorithm (sfFFTs‐NLLS), it provides high‐accuracy quantification of period, amplitude, and phase, benchmarked to established platforms such as BioDare2, with near‐perfect correlation (r >0.99) of period and amplitude values calculated on the same dataset (n = 23). This pipeline allows rapid and reproducible analysis of circadian traces, lowering the technical threshold for time series analysis and promoting open data practices. Through iterative rounds of algorithm implementation, manual refinement, and independent validation, we devised a new sfFFTs‐NLLS algorithm, returning results that are highly consistent with well‐established FFT‐NLLS methods [34]. However, as the sfFFTs‐NLLS algorithm only fits one cosine frequency as opposed to the FFT‐NLLS, it may not be as effective in analyzing complex, changeable waveforms. The full implementation is available on ClockCyteR GitHub repository. The large language model (LLM) ChatGPT 4.0 (OpenAI) was used to brainstorm strategies for period quantification and draft the structure of the period detection algorithm. This output was further developed by the authors to implement an improved algorithm, detecting circadian phase, and was integrated with the rest of the pipeline. An analysis of the same dataset using already established tools (i.e., Biodare2) [35] was performed to verify accuracy. This strategy enabled the implementation of a reliable and efficient period detection strategy for the fast analysis of time series applicable to streamlined high‐content data processing.

In parallel, *ClockCyteR.spatial* enables spatially resolved reconstruction of circadian rhythms in hundreds of ROIs across tissue slices. By mapping rhythmic parameters (e.g., period and phase) across the tissue, this tool provides a powerful framework for examining how intercellular synchrony, activity clustering, and network coherence spatially evolve in unperturbed conditions and under genetic or pharmacological treatments. Overall, the *ClockCyteR* suite establishes a reproducible and extensible computational environment for spatiotemporal circadian analysis, supporting both fundamental and applied research into circadian network organization.

### 3.4 Compartmentalized Calcium Rhythms in the SCN

Intracellular neuronal calcium levels are essential for the physiological regulation of several key aspects of circadian rhythmic behavior, including synaptic plasticity, period determination, and neuropeptidergic release [36]. Intracellular neuronal calcium is highly rhythmic in the SCN, and has been associated with the electrical‐genetic coupling of the SCN via the coordinated activation of CRE‐elements of E‐box‐containing clock gene promoters, critically contributing to SCN tissue synchronization [10, 37]. In line with this view, intracellular neuronal calcium activity and clock gene expression (as measured by the PER2::LUC reporter) are similarly organized, with calcium sweeping through the SCN circuit in highly stereotyped spatiotemporal waves preceding PER2 clock gene expression by about 6 h [10, 38].

However, this apparent consistency may conceal an unexpected complexity to intracellular calcium regulation in the SCN. Clock gene knockout (double Cry‐null) in SCN slices strongly affected the phase relationship between intracellular neuronal calcium and PER2 gene expression, but not the relationship between PER2 and firing rate [39]. On the other hand, treatment with tetrodotoxin (TTX) to block neuronal firing in the SCN only reduces intracellular calcium rhythms by about 30 % [38]. This suggests that two components may exist in the neuronal organization of intracellular calcium within the SCN: one generated as an output from the intracellular clock gene expression (sensitive to the knocking out of the Cry genes), and a second one, inherently dependent on a 24 h synchronizing signal produced by the SCN circuitry [39]. Moreover, different cell types within the SCN can show very different intracellular calcium rhythms: astrocytic calcium is also rhythmic and peaks at nighttime, as opposed to daytime neuronal calcium. Interestingly, astrocytic intracellular calcium is organized in phase‐homogeneous circadian “pulses” as opposed to neuronal phase waves [4, 15, 40].

Electrophysiological recordings by multiunit activity array recordings (MUA) in acute slices return a somewhat dichotomic picture of neuronal activities within the SCN, which appear to be far more synchronized than clock gene expression and intracellular neuronal calcium [41]. Such a dichotomy has also been reported in SCN organotypic cultures, whereby membrane potential is more synchronous than co‐detected intracellular neuronal calcium [15, 25]. Our findings with the ARCLightD reporter in the ClockCyte are consistent with these reports, as they return a more homogenous synchronization of the ARCLightD signal compared to the intracellular reporter Syn‐jRCAMP1a (Figure S5H).

The SCN is characterized by a high number of intranuclear axons, with a majority of (unmyelinated) axons fully comprised within the SCN itself [42, 43], suggesting that axonal calcium signals may be crucially important for within‐SCN circuit synchronization. To image circadian oscillations of axonal calcium, we used the genetically encoded reporter GCaMP6 fused to an axonal subcellular localization peptide from the growth‐associated protein 43 (GAP43), to enrich GCaMP localization in axons (Figure S6) [23, 44]. Axonal calcium oscillations displayed the same ∼24 h periodicity as intracellular neuronal calcium but with significantly sharper waveform profiles, reduced circular variance, and greater spatial homogeneity (Figure 3). This suggests that axonal compartments may integrate or distribute circadian signals with high phase fidelity across the SCN circuitry. Notably, the uniform phase distribution of axonal calcium is in strong contrast with the wave‐like phase gradients of neuronal intracellular calcium, similarly to what was previously shown by electrophysiological recordings and live imaging voltage reporters.

By using the same measuring calcium probe (GCaMP), enriched in different subcellular compartments (axonally‐enriched vs. intracellular), we now confirm that such a dichotomy of temporal signal organization within SCN neurons (pulses vs. waves) is likely to reflect the distinct temporal contributions of membrane‐bound events of electrical origin (i.e. membrane potential, firing, axonal calcium), vs. intracellular events (intracellular calcium, clock gene expression), rather than resulting from technical discrepancies due to different probes (ARCLight vs. GCaMP), experimental preparations (acute vs. organotypic slices), or detection modalities (electrophysiology vs. imaging).

While the available evidence gathered within the SCN organotypic cultures supports such a model, it is important to consider some potential technical limitations when interpreting our findings. A potential limitation of using Axon‐GCaMP6s to monitor axonal calcium dynamics in SCN slices relates to the specificity and interpretation of the recorded signal. Although we confirmed axonal enrichment through particle size analysis and colocalization with the presynaptic marker Synaptophysin 1 (Figure S6), it may not be exclusively restricted to the axonal compartment in the SCN. Residual expression in somatic or dendritic regions, even at low levels, could contribute to the measured fluorescence, particularly when combined with optical factors such as light scattering and pixel‐level averaging inherent to wide‐field live‐imaging of dense neural tissue in thick SCN slices. However, membrane‐bound GCaMP probes localized to the presynaptic compartment of the SCN by a Synaptophysin‐derived peptide do not show a pulse‐like organization in their signal distribution [4], notwithstanding that similar considerations of pixel‐size averaging and light scattering will apply to those signals [44]. Thus, these findings suggest that the pulse‐like organization of the axonal signal is specific and not fully explained by spatial averaging. Moreover, they also suggest that the circadian regulation of the proximal vs. distal axonal compartments may be different; however, future studies of high‐resolution, high‐magnification long‐term live imaging will be needed to untangle finer circadian regulation within SCN axons.

A second limitation to consider is that SCN slice isolation will sever axonal projections to and from the SCN that will degenerate over several weeks of culturing; crisscrossing intra‐SCN axons may also be damaged, which is likely to trigger some structural reorganization within the tissue over time. Nevertheless, SCN synaptic connectivity is retained and essential within SCN organotypic cultures, as shown by the Tetrodotoxin (TTX) blockade of voltage‐gated sodium channels disrupting tissue‐level circadian synchronization of neuronal calcium activity and clock gene expression, while preserving single‐cell oscillations [38, 45, 46]. Consistently, we have recently shown that selective disruption of presynaptic vesicular release in SCN organotypic cultures (by TeLC‐mediated cleavage of VAMP2) similarly reduces circadian rhythm amplitude and clock gene synchronization [4]. Moreover, circadian circuit synchronization recovers after TTX washout, indicating that the remaining synapses within SCN slices are sufficient to autonomously re‐establish circadian coherence despite the initial severing and reduction. As the vast majority of SCN synapses contain axons, they are likely to contribute to such presynaptic signaling. However, a small fraction of dendro‐dendritic synapses (DDCSs) has been reported in the SCN, with ∼10.9 % of dendrites in the core and 1.9 % of synapses in the shell forming at least one dendro‐dendritic chemical synapse [42, 43]. Thus, organotypic cultures are likely to return only a partial perspective on SCN activities; nevertheless, they remain a very powerful model to inform future in vivo experimentation, as more refined molecular tools and detection systems become available.

### 3.5 Network‐Level Temporal Information Stored in Axons is Degraded by Bmal1 Deletion

Bmal1 is the only non‐redundant circadian clock gene in mammals. While its essential role in circadian rhythms in single cells is well established, less clear is its role as a molecular transducer of circadian temporal information through brain circuits. Not only is Bmal1 an essential regulator of axonal regeneration [48], but it is also rhythmically expressed in synapses, with rhythmic phosphorylation at key Ser residues respectively modulating its translocation to the cytosol, or association with Calmodulin Kinase 2α (CaMKIIα), an essential modulator of synaptic function and plasticity [48, 49]. Thus, we investigated the effects of Bmal1 deletion on axonal calcium as a readout for SCN circuit organization. Thanks to the newly developed Clockcyte platform, we were able to monitor the dynamic changes in the circuit organization following Bmal1 ablation in neurons. While analysis of mean circadian rhythms suggested full abolition of circadian rhythms in the SCN (Figure 4A), more detailed tissue‐wide analysis of single neuronal oscillators within the SCN circuit returned a more complex picture. We observed that ablating Bmal1 in neurons did not fully ablate circadian oscillations of axonal calcium within the SCN, albeit strongly reducing them (up to 30 % remaining axonal oscillators were found, even after more than 10 days from the ablation) (Figure 4D,E). Interestingly, neuronal Bmal1 ablation did not alter the mean period specified by axonal calcium (Figure 4F) but reduced the accuracy of the periodic information through the circuit (Figure 4G,H). This suggested that Bmal1 ablation in neurons may primarily impair the neuronal period consistency across the SCN circuit, and the overall abolition observed in the mean traces may be due to a weakened period coherence through the SCN circuit, rather than a full abolition of circadian function. Notably, accelerated PER2::LUC rhythms were detected in SCN slices (∼18 h) following constitutive germline deletion of Bmal1 [50], which we did not detect in our experiments. Key differences exist between the two models: we use postnatal, neuron‐specific somatic viral deletion, whereas germline knockout ablates Bmal1 in all cells and already during development. Preservation of the TTFL in astrocytes, as well as developmental effects in Bmal1 constitutive knockout, may both contribute to the differences observed across the two models. To investigate the network underpinnings of the observed alterations, we developed two separate analysis pipelines within ClockCyteR.spatial (cluster structure and period coherence analysis). Cluster structure analysis revealed that while homogenous in phase, the axonal Ca^2+^ is organized in discrete local clusters (Figure 5A). The reciprocal interactions amongst clusters are impacted soon after Bmal1 ablation, before the internal coherence measured by intra‐cluster phase variance and the strength of the correlation coefficient is altered in the late stages (Figure 5B–D). This suggests that longer‐range axonal connectivity within the SCN may be more vulnerable to circadian clock disruption than local axonal connectivity. The increased intermingling of the clusters was accompanied by an early reduction of periodic coherence across the SCN circuit (Figure 5E–G), suggesting that the reduced spatial segregation of the axonal calcium cluster may be rooted in a reduced period coupling across the SCN circuitry. Overall, our findings show that Bmal1 is essential for maintaining the stability and coupling of axonal rhythmicity across the SCN tissue. The observed dampening and abolishment of emerging mean circadian rhythms is due to the progressive weakening of the inter‐cluster network structure associated by reduced period coupling. Altogether, these findings link clock gene integrity to the maintenance of precise temporal coordination across brain tissue networks, emphasizing the importance of circuit‐level mechanisms for circadian homeostasis.

### 3.6 Broader Applications

Importantly, ClockCyte imaging is not restricted to the SCN or to the brain: its modular design can be adapted to other neural circuits and to non‐neural tissues that retain intrinsic rhythmic and/or network properties in culture. It will provide a versatile platform to investigate long‐term, circuit‐level dynamics in intact tissue, as well as tissue‐wide and therapeutic responses across diverse experimental contexts. Its capacity for stable, multiplexed imaging over weeks makes it particularly well suited for ex vivo models of neurodegeneration, whereby progressive alterations in neuronal, glial, and network function central to disease pathogenesis could be tracked in real time. Organotypic cultures from models of Alzheimer's disease, Parkinson's disease, or related dementias could be used to longitudinally track functional decline, circuit remodeling, and cell‐type‐specific vulnerability in response to disease initiation under defined conditions [51, 52, 53]. Similarly, intra‐tissue neoplastic and metastatic processes could be tracked for several weeks to evaluate the contribution of different cells and microenvironmental factors in the tissue parenchyma [54]. Finally, the platform will enable systematic assessment of drug treatments, chronotherapeutic strategies, or disease‐modifying compounds across multiple tissues in ex vivo models.

## 4 Methods

### 4.1 Animal Work

All animal procedures were conducted in accordance with the UK Animals (Scientific Procedures) Act 1986 and approved by the UK Home Office under Project Licence (PPL) number [PP5265525] awarded to Brancaccio. Mice were housed in a 12:12 light‐dark schedule and fed ad libitum. Mouse strains used were PER2::LUC (B6.129S6‐Per2tm1Jt/J), RRID: IMSR_JAX:006852 [22], a gift from Dr Michael Hastings, MRC‐LMB, Cambridge, UK, and BMAL1Flox (allele B6.129S4(Cg)‐Bmal1tm1Weit/J) [55], a gift from Professor Simone di Giovanni (Imperial College London).

### 4.2 SCN Organotypic Slice Preparation

SCN organotypic slices were prepared as previously described [11]. Briefly, brains from p11‐p15 mouse pups were extracted after cervical dislocation and dissected on ice while immersed in dissection medium (GBSS [G9779, Sigma] with 5 mg/mL glucose [158968, Sigma], 100 nM MK801 [M107, Sigma], 3 mM MgCl_2_ [AM9530G, Invitrogen], 50 µM AP‐5 [0106, Tocris] filtered with 0.22 µm pore size Steriflip). Three cuts were manually performed to isolate the medial‐ventral part containing the SCN, and coronally sliced at 300 µm by using a tissue chopper (McIlwain). Using a light microscope, slices containing the SCN were isolated and placed on a membrane (PICM0RG50 or PICM01250, Millipore), and incubated in initial plating medium (HEPES‐buffered “air medium”, supplemented with 100 nM MK801, 3 mM MgCl2 and 50 µM AP‐5; “air medium”: 500 mL ddH2O with 4.15g DMEM [D5030, Sigma], 0.175g NaHCO_3_, 2.25g glucose [158968, Sigma], 5 mL penicillin/streptomycin [P4333, Sigma], 5 mL HEPES 1 M [H0887, Sigma], 5 % horse serum [10270106, Gibco], 1 % B27 [17504044, Gibco] and 0.5 % Glutamax [35050‐038, Invitrogen]) for 2–4 h before being transferred to air medium and sealed with plate film (Z369667, Excel Scientific). All samples were left to settle for at least 6 days before treatment, and the medium was changed once a week.

### 4.3 AAV Transduction

SCN slices were transduced by adding 1 µl of AAV suspension on top of the slice and allowing the expression for no less than 48 h before changing the medium. AAVs used in this study were: hSyn‐NES‐jRCaMP1a‐WPRE‐SV40 (Douglas Kim; 100848, AAV1, 1.9 × 10^13 vg/mL), hSyn‐iGABASnFR (Loren Looger; 112159, AAV1, 2.1 × 10^13 vg/mL), hSyn‐jGCaMP8s‐WPRE (162374, AAV1, 1.9 × 10^13 vg/mL), hSyn‐axon‐GCaMP6s (Lin Tian, 111262, AAV1, 1.8 × 10^13 vg/mL), hSyn‐Cre (Johan Jakobsson, 170367, AAV1, 2.0 × 10^13 vg/mL) from Addgene, and hSyn‐ARCLightD (Vincent Pieribone, AV‐1‐36857P, AAV1, 1.9 × 10^13 vg/mL), from the Penn Vector Core. For reporter vectors, the transduction was performed at least 7 days before the start of the recording. When transducing with multiple reporters, the transductions were done sequentially, leaving at least 7 days in between.

### 4.4 Multi‐Channel Long‐Term Live Imaging of SCN Slices

Multichannel long‐term live imaging of SCN slices was performed using either the Sartorius Incucyte S3 system or the Olympus (Evident Scientific) LV200 system.

### 4.5 Sartorius Incucyte S3

The Incucyte S3 (Sartorius) system was housed inside a 200 L incubator, and the temperature was set at 36.5°C to prevent additional heat buildup from microscope scanning. Images were acquired using a 4× magnification objective (2.82 µm/pixel), with 300 and 400 ms exposure times for green and red channels, respectively. The samples were focused using the autofocus microscope function. Ex/Em spectra: Green channel (441–481 nm, 503–544 nm); Red channel (567–607 nm, 622–704 nm). Predicted scanning times: fluorescent channels: red, green. Exposure time 300–400 ms; 2 min for each 24‐well. Total scanning time for six 24‐well plates:12 min. Images were acquired every 30 min. SCN slices were imaged in plastic 6‐well plates (734–2777, VWR) or plastic 24‐well plates (83.3922, Sarstedt) with 320 µL medium in each well. A plastic film (Z369667, Excel Scientific) was used to seal the plate, and 300 µL sterile PBS was added to the space between all wells to reduce evaporation and facilitate heat dispersal.

### 4.6 Olympus LV200

Live imaging in the LV200 was performed when acquiring signals from bioluminescent probes (i.e., PER2::LUC). Images were acquired at a 30 min interval using a 40x long‐distance objective. Using a TOKAII HIIT system, the temperature was kept at 37°C, with the top plate set at 38°C to prevent condensation on the upper portion of the plate. SCN slices were imaged in a glass‐bottom 24‐well plate (CellVis #P24‐1.5H‐N) sealed with a plastic film (Z369667, Excel Scientific). For the imaging of PER2::LUC reporter, 100 µM Luciferin (AAT Bioquest #12505) was added to the culture medium, and images were acquired with a 2 min exposure time.

### 4.7 Immunofluorescence on SCN Slices

SCN slices were fixed for 1 h in 4 % PFA/PBS at room temperature. For immunofluorescence, tissue was incubated for 1 h in Day1 buffer (1X PBS, 1 % bovine albumin, 0.3 % Triton X‐100) with 10 % donkey or goat serum at room temperature. Slices were subsequently incubated with primary antibody against either BMAL1 (NB100‐2288, Novus Biological) or Synaptophysin 1 (101‐006, Synaptic Systems) overnight at 4°C. SCN slices were washed twice for 10 min in Day 2 buffer (1:3 dilution of Day 1 buffer in PBS) and incubated overnight at 4°C with a 1:1000 dilution of either donkey anti‐rabbit Alexa Fluor 546 (A10040, Invitrogen), goat anti‐chicken Alexa Fluor 488, 546, or 647 (A‐11039, Thermofisher; A11040, Invitrogen; 703‐606‐155, Jackson Immunoresearch, respectively). After 2 × 5 min washes in Day2 buffer, and 3 × 20 min washes in PBS, samples were mounted on a glass slide with mounting medium containing NucBlue (P36981, Invitrogen). Slices were imaged at 40x (Oil immersion) on a confocal microscope (Leica SPi8) to assess BMAL1 expression.

### 4.8 Data Analysis

#### 4.8.1 Time Series and Circadian Analysis

Time series of mean overall SCN intensity were detrended using a third‐order polynomial in Graphpad Prism v10. Time series analyzed in Figures 1, 2F–G, and 3A, and Figure S2E–H, were imported into Biodare2 [20] for period analysis.

#### 4.8.2 Immunofluorescence Image Analysis

Semi‐automated quantification of BMAL1 protein levels was performed using a custom macro in ImageJ/FIJI. In brief, ROIs were manually drawn around the SCN nuclei. The integrated density of the signal in the BMAL1 channel was extracted using a mask based on the intersection of NucBlue (DAPI) and the inverse of the Axonal‐GCaMP6s signal to identify the neuronal nuclei. Pixel‐based colocalization analysis was performed using the JACoP colocalization plugin in FIJI.

#### 4.8.3 ClockCyteR

The analysis of whole SCN slices time series extracted from Incucyte software was done using the R package ClockCyteR (https://github.com/cabaJr/clockcyteR). The list of packages used to develop the package is available in Table S1. In brief, time series were extracted via the Incucyte microscope control software (Incucyte 2022B Rev2) using the in‐built analysis (basic analyzer, top‐hat segmentation) and imported for analysis using ClockCyteR GUI. Launching ClockCyteR (clockcyteR::run_app()) produces a Shiny [19] web app, allowing the interactive upload of raw .txt files. The imported data from each sample is assigned to an individual object containing information about the experimental condition, and the user can preprocess the signal to perform outlier exclusion and linear/polynomial detrending. Period analysis is available using the Χ^2^, Autocorrelation (AC), Lomb–Scargle, and Continuous Wavelet (CWT) algorithms from the zeitgebr package [21]. Additionally, a single‐frequency Fast Fourier Transform‐seeded‐NonLinear Least Squares (sfFFTs‐NLLS) algorithm was implemented to compute circadian features of the time series, including period length, amplitude, phase, and relative amplitude error (RAE, calculated on a single frequency). The analysis can be restricted to specific time windows or a subset of the dataset. Individual traces or cumulative plots can be generated, including plotting of circadian features, divided by experimental group. Plots and data tables are available for export in .png, .svg, or .csv.

#### 4.8.4 Period Estimation

Period estimation was performed using an implementation of the FFT‐NLS algorithm in the R programming language. We named this function sfFFTs‐NLLS (single‐frequency FFT‐seeded‐NLLS). The algorithm has two main steps.

Each time series is initially transformed in the frequency domain using the discrete Fourier transform (*FFT*) function from the *stats* R package (*stats::FFT*). From the resulting spectrum of signal amplitudes at different frequencies, the dominant one (*f_dom_*) is identified, and the corresponding initial period estimate is calculated as *P*_0_ =  1/*f_dom_*.

The time series *y*(*t*) is then modelled as a cosine function:

(1) $$\begin{array}{c} y\;\left(t\right)=A\cdot \sin\left(\frac{2\pi t}{P}+\phi \right)+C \end{array}$$

where *A* is the amplitude, *P* is the Period, ϕ is the phase, and *C* is the offset. The parameters are optimized using the Levenberg‐Marquardt algorithm implemented in the *nls.lm* function from the *minpack.lm* R package (*minpack.lm::nls.lm*). The FFT‐estimated period *P*_0_ is used as an initial period estimate, and a 16–32 h period range was imposed to match expected circadian ranges. The function implementation is available in the ClockCyteR repository on GitHub (https://github.com/cabaJr/clockcyteR). Parts of the algorithm development, including brainstorming approaches and iterative refinement, were assisted by ChatGPT (OpenAI, GPT‐4 model). All code outputs were reviewed, verified, and adapted by the authors.

#### 4.8.5 Spatiotemporal Circadian Activity Analysis–ClockCyteR.Spatial

A grid creating 5 × 5 pixel ROIs was overlaid on the image stack. ROI coordinates and the time series values containing the fluorescent signal from each ROI were extracted for each channel using a set of custom FIJI macros. Data were imported into R for further processing. To reconstruct the spatial location of each ROI, a custom script was used to combine the grid coordinates with the circadian analysis of the time series data. Processing of ROI coordinates was done using the sf package [56]. Plotting of the data and generation of spatial maps representing the circadian analysis were performed using the ggplot2 package [57].

The code of the FIJI macros and the R package scripts are available in the ClockCyteR.spatial repository on GitHub (https://github.com/cabaJr/ClockCyteR.spatial). The list of packages used to develop the package is available in Table S1.

#### 4.8.6 Network Analysis

Coherence analysis was performed on combining the period analysis results with the grid spatial coordinates. For each ROI in the grid, defined by a pair of coordinates *x_i_* =  (*X_i_*, *Y_i_*), the parameter of interest *V* (i.e., period length) was compared across the set of ROIs *N_i_* (*r*) within a specified radius *r*, measured by the Euclidean distance of ROI coordinates. The difference between the two parameters |*V_i_* − *V_j_* | was calculated, and the fraction of “coherent” ROIs *C_i_* (*r*, *t*) was defined as the number of ROIs in the defined area whose difference was within a specified threshold *t*.

(2) $$\begin{array}{c} C_{i}\;\left(r,\;t\right)=\left\{\;j\in N_{i}\left(r\right)\;|\;\left|V_{i}-V_{j}\right|\leq t\right\} \end{array}$$

The coherence ratio (*CR_i_*) was calculated accordingly:

(3) $$\begin{array}{c} \;CR_{i}=\frac{C_{i}\left(r,\;t\right)}{N_{i}\left(r\right)} \end{array}$$

To identify clusters of ROIs in SCN slices the following method was used: an all‐to‐all correlation of axonal Ca^2+^ traces was conducted using an unsupervised, combined approach, weighting Spearman correlation (rank‐based, sensitive to phase shifts), FFT spectrum analysis identifying period and amplitude of traces (frequency‐based, robust against noise), and a distance weight decreasing the correlation score of ROIs far from each other. The trace correlation matrix was transformed using an arctangent function to highlight differences at the tail of the distribution. A threshold was set to isolate the top 10 % of the distribution. The R package *igraph* [58] was used to perform network analysis and community detection. The Leiden algorithm, from the igraph package, was used to identify clusters (n_iterations = 200, resolution = 0.75). Clusters comprising less than 3 ROIs were deleted, and the respective ROIs were assigned to a “Null” cluster. Clusters were ordered based on the main phase of the rhythmic ROIs belonging to it. The segregation between clusters was calculated pairwise as the fraction of overlapping area between each convex hull representing a cluster. The segregation index is calculated as the mean between cluster segregation values weighted by the number of ROIs in each cluster. The code is available in the ClockCyteR.spatial repository on GitHub (https://github.com/cabaJr/ClockCyteR.spatial).

### 4.9 Image Processing

Preparation of raw imaging files from Incucyte was done in ImageJ/FIJI [59], using a set of in‐house‐made macros (https://github.com/cabaJr/Incucyte_processing), including image stitching, registration, and rotation. Stacks were registered using the description‐based series registration 2d/3d+t plugin (Movie S3) (https://github.com/fiji/Descriptor_based_registration). The SCN boundaries for mean or ROI (grid‐based) signal extraction were identified using manually drawn regions of interest (ROIs).

### 4.10 Statistical Analysis

Statistical analysis was performed in GraphPad Prism v10 or R v 4.4.2 (Figures 3I, 4G, and 5G). Manual drawing of ROIs was performed blind to the sample genotype. Non‐parametric statistical analysis was performed on datasets failing the Shapiro–Wilk normality test. All statistical comparisons are specified in the figure legend. Statistical comparisons involving two groups were conducted using paired, unpaired, or nested *t*‐tests, as specified. Multiple time points comparisons between two groups were performed using a two‐way ANOVA with Tukey's or Šídák's multiple comparison. Kernel density estimation of the ROI period and phase distribution was calculated and plotted using the ggplot2 package in R [57]. 2‐sample Kolmogorov–Smirnov test was performed using the matrixStats package in R [60]. The Rayleigh test of uniformity was calculated using the circular package in R [61]. The Wasserstein distance between two vectors (Figure 4G) was calculated using the transport package in R [62].

## Author Contributions

Conceptualization: Marco Ferrari, Natalie Ness, and Marco Brancaccio; Methodology: Marco Ferrari, Natalie Ness, and Marco Brancaccio; Investigation: Marco Ferrari, Natalie Ness and Julieta Acosta; Visualization: Marco Ferrari; Funding acquisition: Marco Brancaccio; Project administration: Marco Ferrari and Marco Brancaccio; Supervision: Marco Brancaccio; Writing – original draft: Marco Ferrari and Marco Brancaccio; Writing – review and editing: Marco Ferrari, Natalie Ness, Marco Brancaccio, and Julieta Acosta.

## Funding

The funding was supported by the UK Dementia Research Institute award number UKDRI‐5007 through UK DRI Ltd (MB) and Michael Uren Foundation (MB).

## Conflicts of Interest

The authors declare no conflicts of interest.

## Supporting information




**Supporting File 1**: advs75427‐sup‐0001‐SuppMat.docx.

**Supporting File 2**: advs75427‐sup‐0002‐MovieS1‐S3.zip.

## Data Availability

The ClockCyteR and ClockCyteR.spatial packages are currently deposited on GitHub repositories (https://github.com/cabaJr/ClockCyteR) (https://github.com/cabaJr/ClockCyteR.spatial). The raw data used in the generation of the figures are available upon request.
