# Supplementary material for: A High‐Throughput Live Imaging Platform to Investigate Circuit‐Dependent Regulation of Circadian Rhythms in Brain Tissue
Source: Adv Sci (Weinh). 2026 Apr 28;13(40):e75427. doi: 10.1002/advs.75427 (PMC13335426; doi:10.1002/advs.75427)
Supplement: Supplementary file 1 — Supporting File 1: advs75427‐sup‐0001‐SuppMat.docx. [file ADVS-13-e75427-s001.docx]

Supporting Information

A high-throughput live imaging platform to investigate circuit-dependent regulation of circadian rhythms in brain tissue

Marco Ferrari^1,2^, Natalie Ness^1,2,†^, Julieta Acosta^1,2^, Marco Brancaccio^1,2^*

^1^ UK Dementia Research Institute at Imperial College London, London, United Kingdom

^2^ Department of Brain Science, Imperial College London, London, United Kingdom

^†^ Current address: The Francis Crick Institute, London, United Kingdom.
E-mail: m.brancaccio@imperial.ac.uk

**Figures and Tables**

**
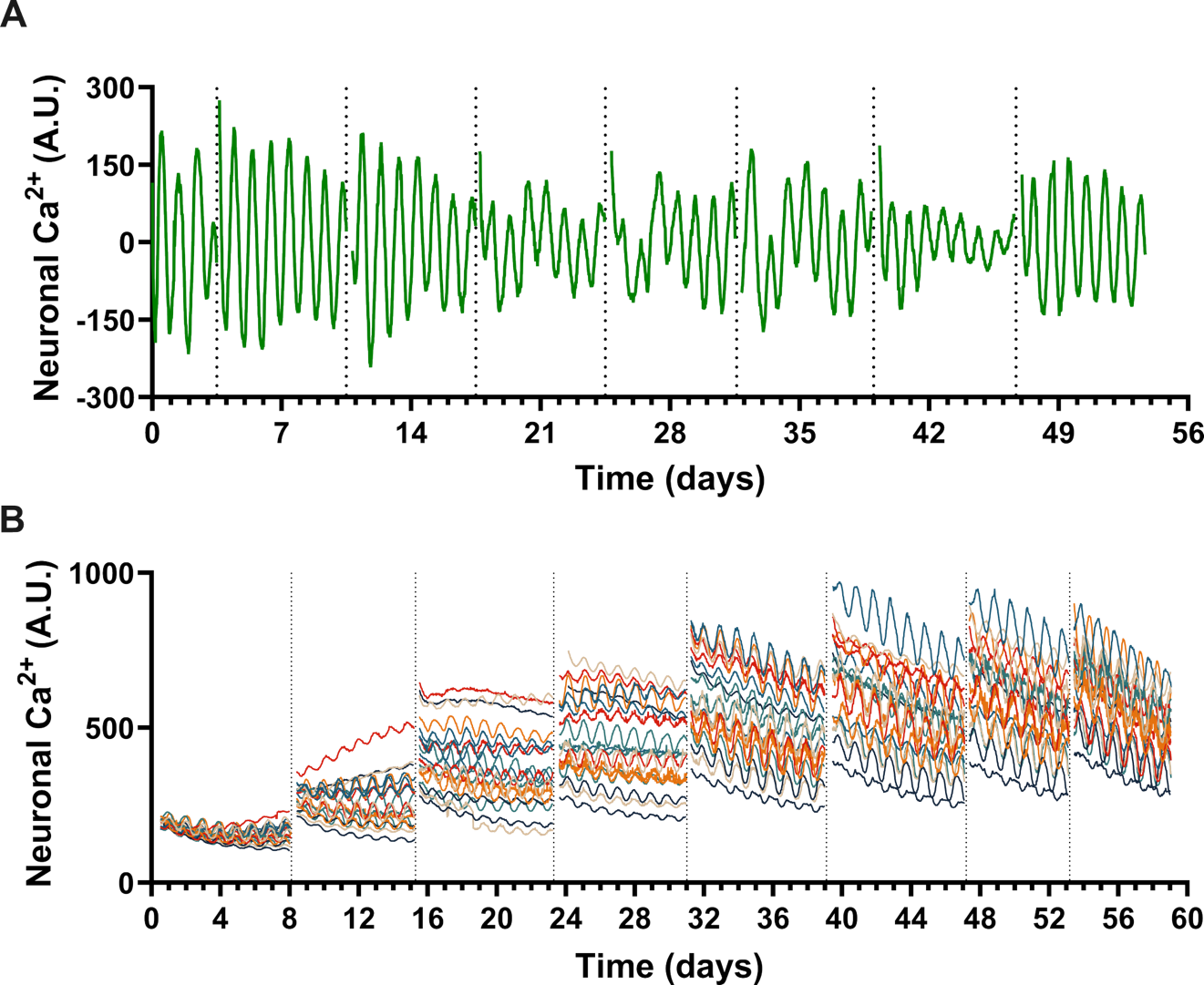
**

**Figure S1. Long-term live imaging of circadian oscillations of neuronal calcium in SCN slices**

**(A)** Neuronal Ca^2+^ trace (Syn-jGCaMP8s, detrended) continuously recorded from a single SCN slice. Vertical dotted lines represent medium changes. **(B)** Neuronal Ca^2+^ traces (Syn-jGCaMP8s, raw signal) continuously recorded from a SCN slices cultured in a single 24-well plate.


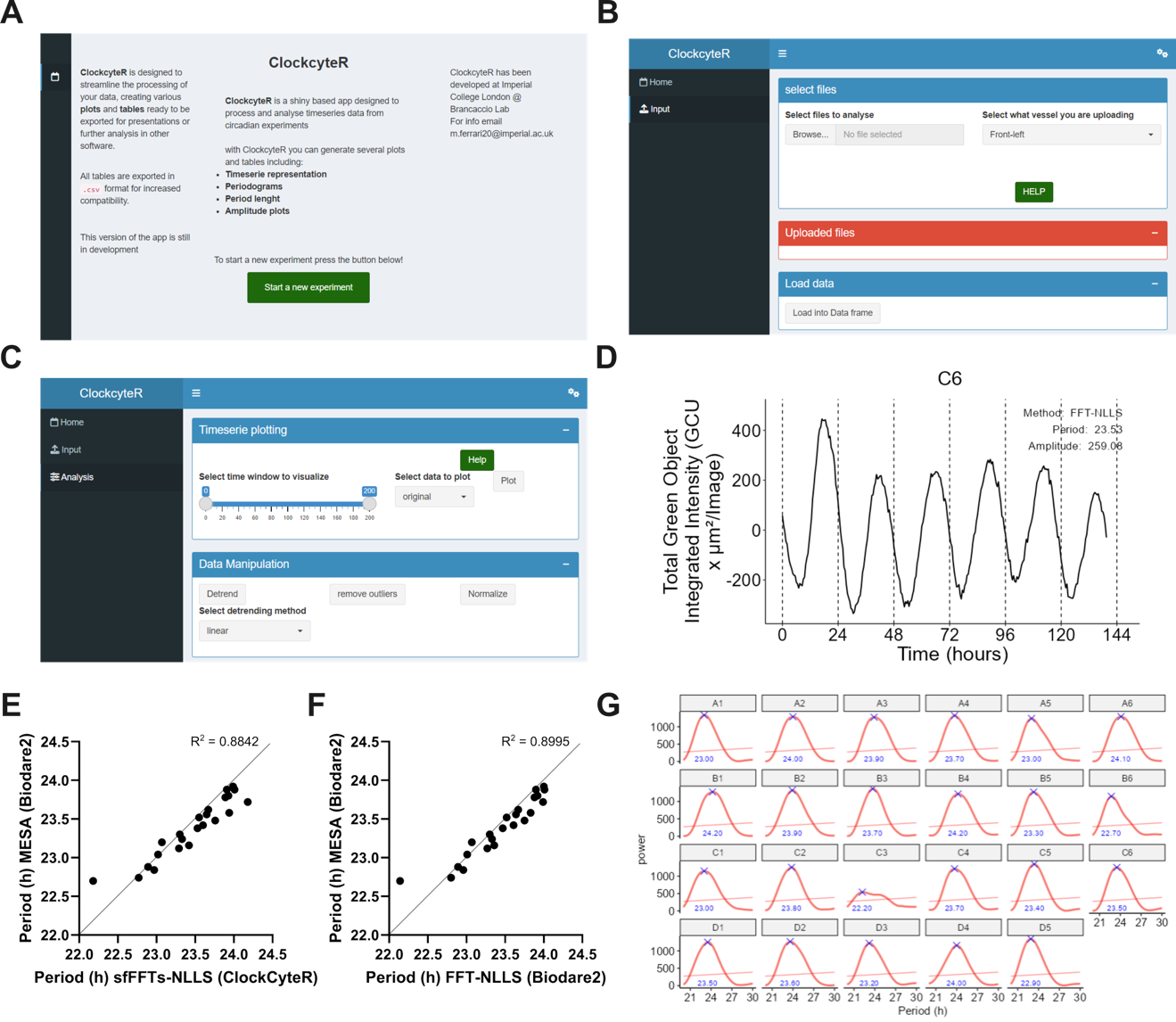


**Figure S2. Establishment of an open-access pipeline for the multivariate wave analysis output from the ClockCyte platform**

**(A)** App interface; landing page. **(B)** App interface; data upload page where the files user selects and uploads the filed used in the analysis. **(C)** App interface: Analysis page, where data are visualised and parameters for the analysis are set (detrending, rhythm analysis, method selection). **(D)** Example trace as visualised in the app. Parameters from the rhythm analysis are automatically overlaid on the graph, and show the method for rhythm detection, the period length and amplitude. **(E)** Correlation between ClockCyteR sfFFTs-NLLS and Biodare2 MESA period analysis, R^2^=0.8842. **(F)** Correlation between Biodare2 MESA and Biodare2 FFT-NLLS period analysis, R^2^ = 0.8995. **(G)** Periodogram of each individual sample obtained using the Lomb-Scargle (LS) algorithm. Rhythm quantification in each sample is represented as a red curve. The straight line indicates the significance threshold.


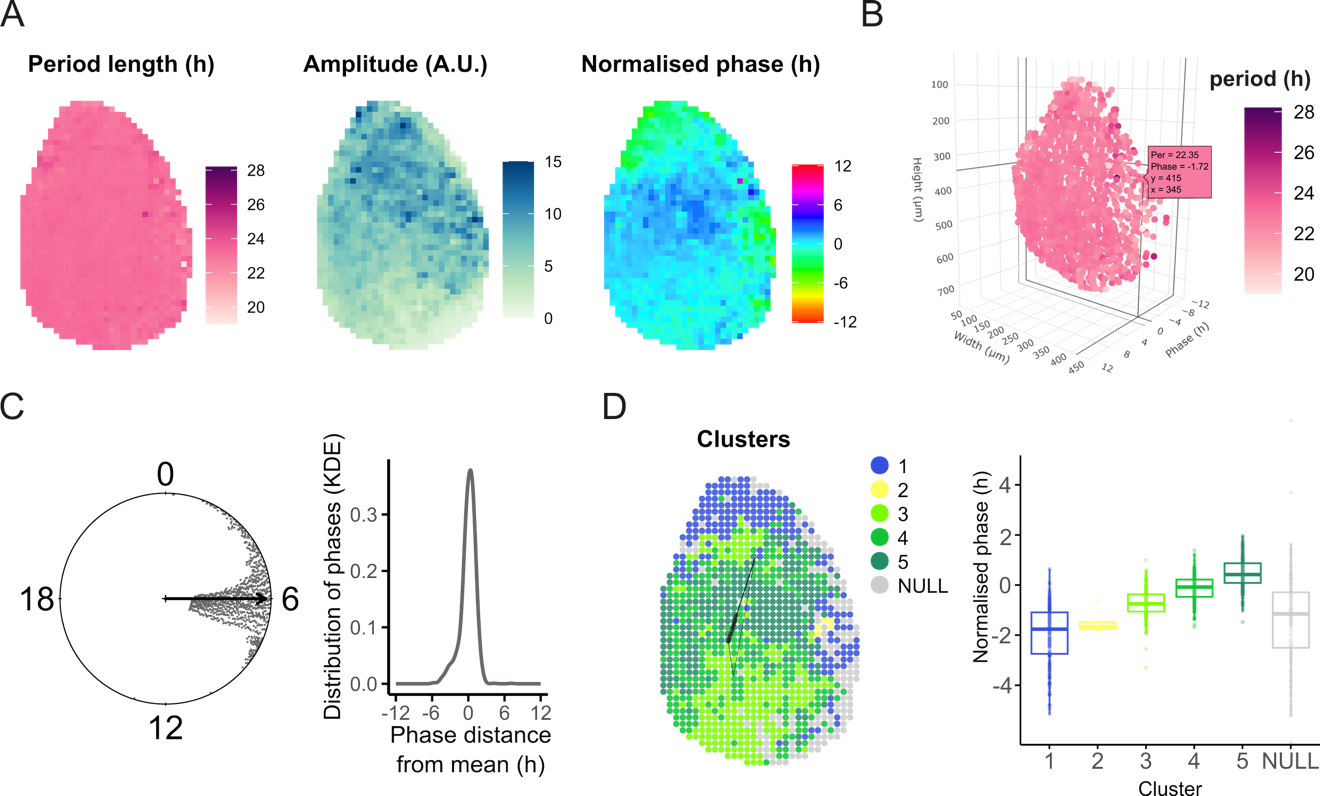


**Figure S3. ClockCyteR.spatial: a pipeline for the spatiotemporal analysis of circadian rhythms**

**(A)** Spatial representation of circadian features of neuronal Ca^2+^ rhythms (Syn-jGCaMP8s): period length, amplitude, and normalized circadian phase. **(B)** Visualization of phase (x axis) and period length (colour coded) in a three-dimensional space. The y and z axis represent the location of each cell in the sample. **(C)** Rayleigh plot representing distribution of ROIs phases of Neuronal Ca^2+^ (left); Distribution (KDE) of phase distances from the mean phase (right). **(D)** Cluster analysis of neuronal Ca^2+^ activity (left); normalized mean phase (h) of each cluster. All panels represent data from the same sample, expressing Syn-jGCaMP8s.


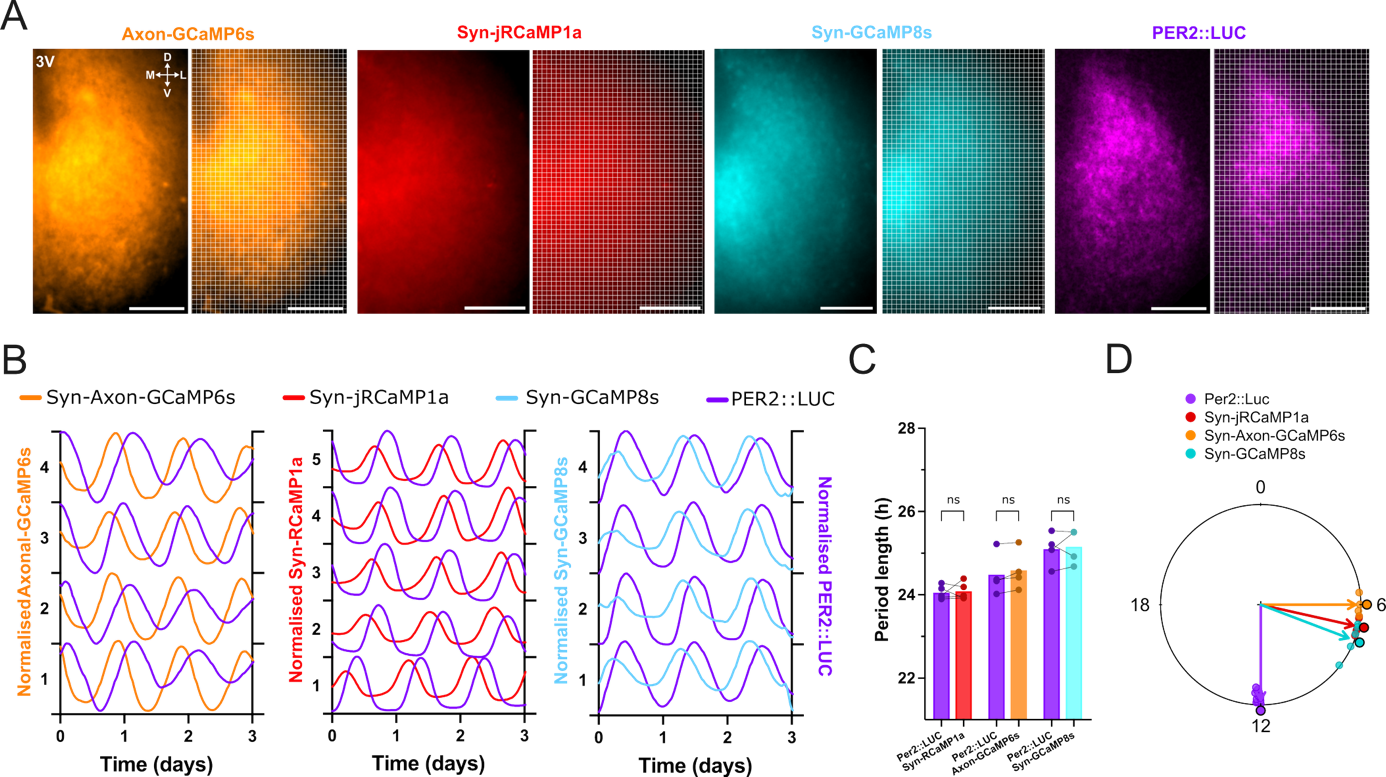


Figure S4. Characterization of circadian expression of Ca^2+^ reporter activity in relation to clock gene expression in SCN slices.

(A) Still image of each reporter at the peak of activity in representative SCN slices, and a superimposed grid to perform ROI analysis. From left to right: Syn-Axon-GCaMP6s, Syn-jRCaMP1a, Syn-GCaMP8s, PER2::LUC. Scalebar=150μm. (B) Traces of PER2::LUC (purple), and neuronal Ca^2+^ from SCN slices transduced with either Syn-Axon-GCaMP6s (orange, left, n=4), Syn-jRCaMP1a (red, middle, n=5), or Syn-GCaMP8s (cyan, right, n=4). (C) Period analysis of SCN slices shown in panel B. Paired one-way ANOVA with Šídák's multiple comparisons test. (D) Phase analysis of traces shown in panel B. Values aligned to PER2::LUC peaks (CT12). Mean values are displayed as points outside the Rayleigh plot. ns=p>0.05.


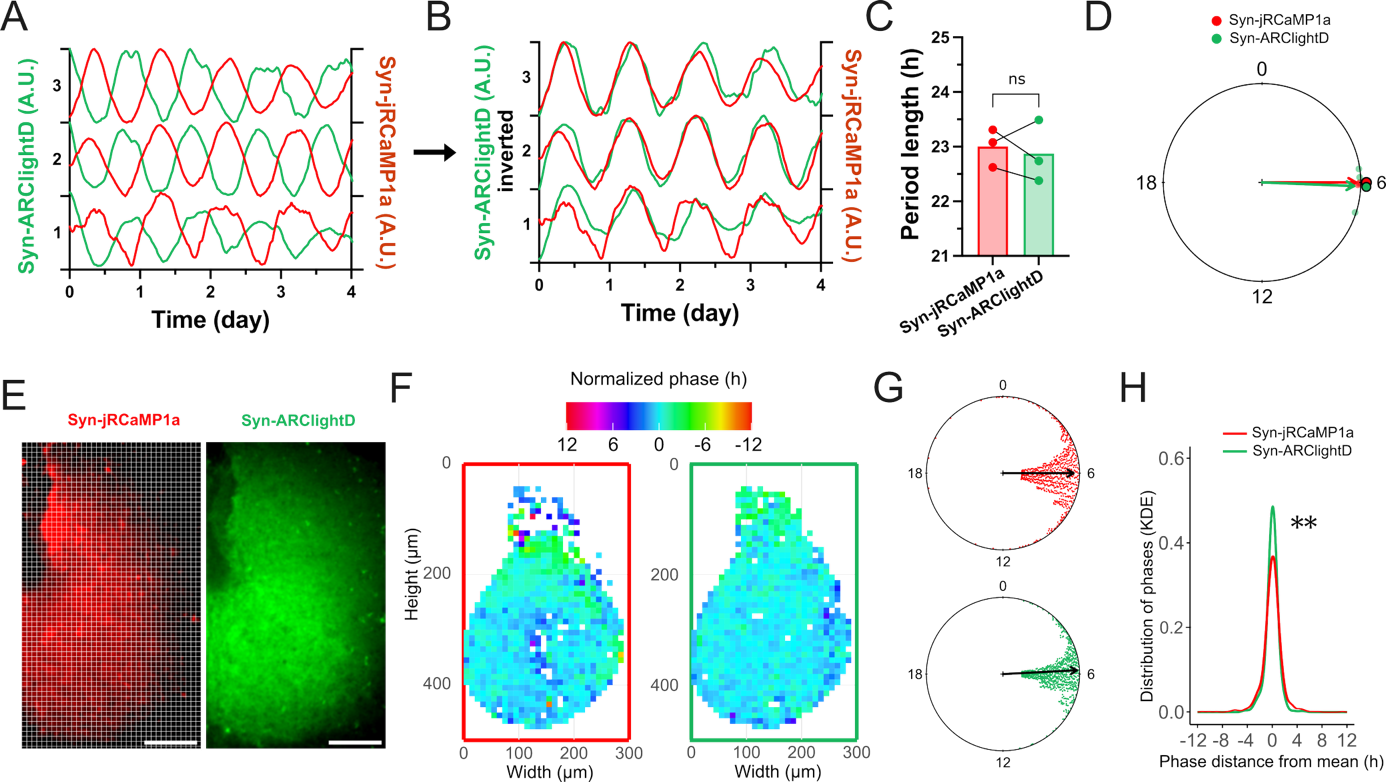


Figure S5. Study of neuronal membrane potential in relationship to intracellular neuronal Ca^2+^ circadian rhythms in the SCN

**(A)** Normalized traces of Syn-ARC-lightD (green, left axis) and Syn-jRCaMP1a (red, right axis) (n=3). **(B)** Normalized traces of Syn-ARC-lightD (inverted) and Syn-jRCaMP1a. **(C)** Period analysis of traces shown in panel B. **(D)** Phase analysis of traces shown in panel B. Syn-jRCaMP1a phases are aligned to CT6. **(E)** Representative images of Syn-jRCaMP1a and Syn-ARClightD co-expression in SCN organotypic slices. Scalebar=150um. **(F)** Spatial maps of normalized phase distribution (phases centred on 0) in Syn-jRCaMP1a (red border, left) and Syn-ARClightD (green border, right). **(G)** Representative Rayleigh plot from ROI analysis of Syn-jRCaMP1a and Syn-ARC-lightD. **(H)** Comparison of phase distribution (KDE) between Syn-jRCaMP1a and Syn-ARClightD (786 and 855 cells, respectively. Kolmogorov-Smirnov test). **=p<0.01, ns=p>0.05.


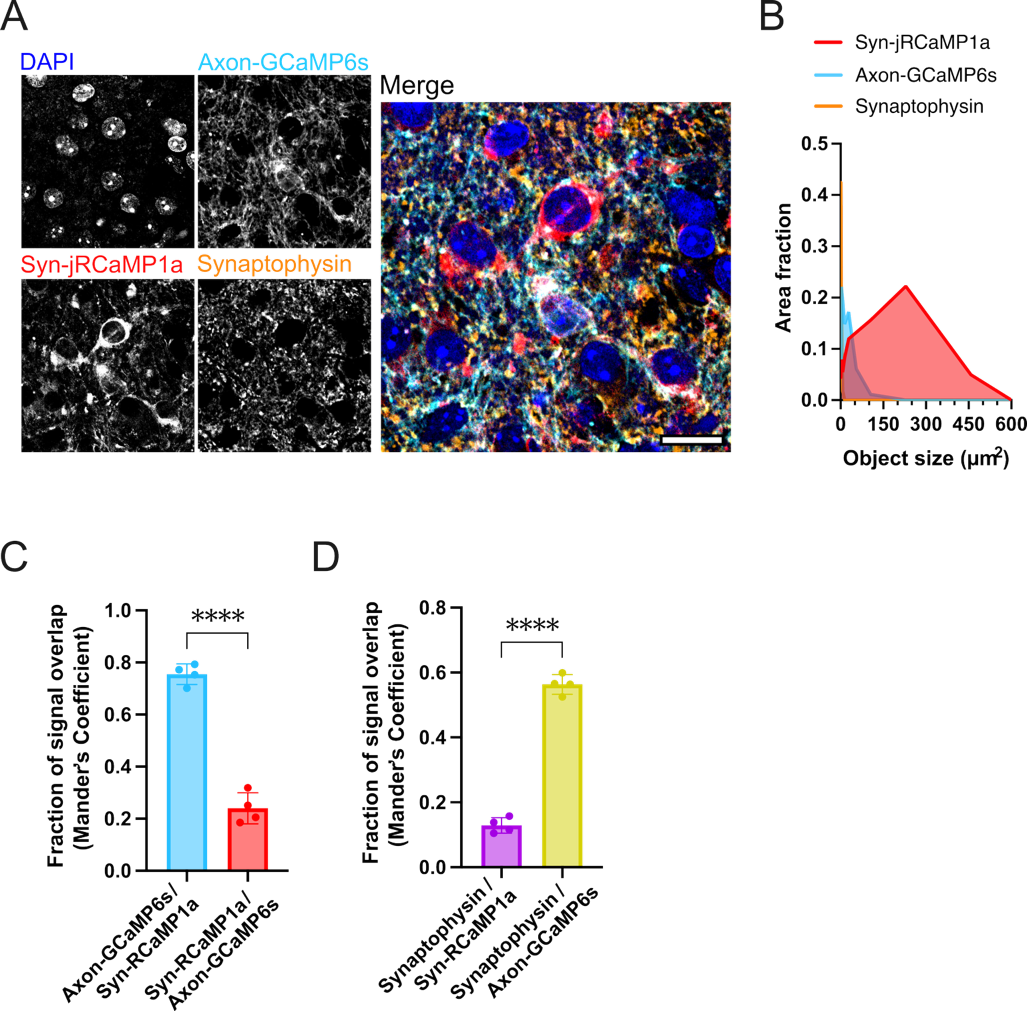


Figure S6. Characterization of subcellular localization of axonal and intracellular Ca^2+^ reporters

**(A)** Confocal images of SCN slice co-transduced with Syn-Axon-GCaMP6s and Syn-jRCaMP1a counterstained with anti-Synaptophysin 1 antiserum. Scalebar=15μm. **(B)** Quantification of area fraction vs object size of signal in Syn-jRCaMP1a, Syn-Axon-GCaMP6s, and Synaptophysin 1 channels. **(C)** Colocalization between Syn-Axon-GCaMP and Syn-jRCaMP1a signal (n=4, Mann-Whitney test). **(D)** Colocalization of Synaptophysin 1 - Syn-jRCaMP1a (left) and Synaptophysin - Syn-Axon-GCaMP signal (right) (n=4, Mann-Whitney test). ****=p<0.01.


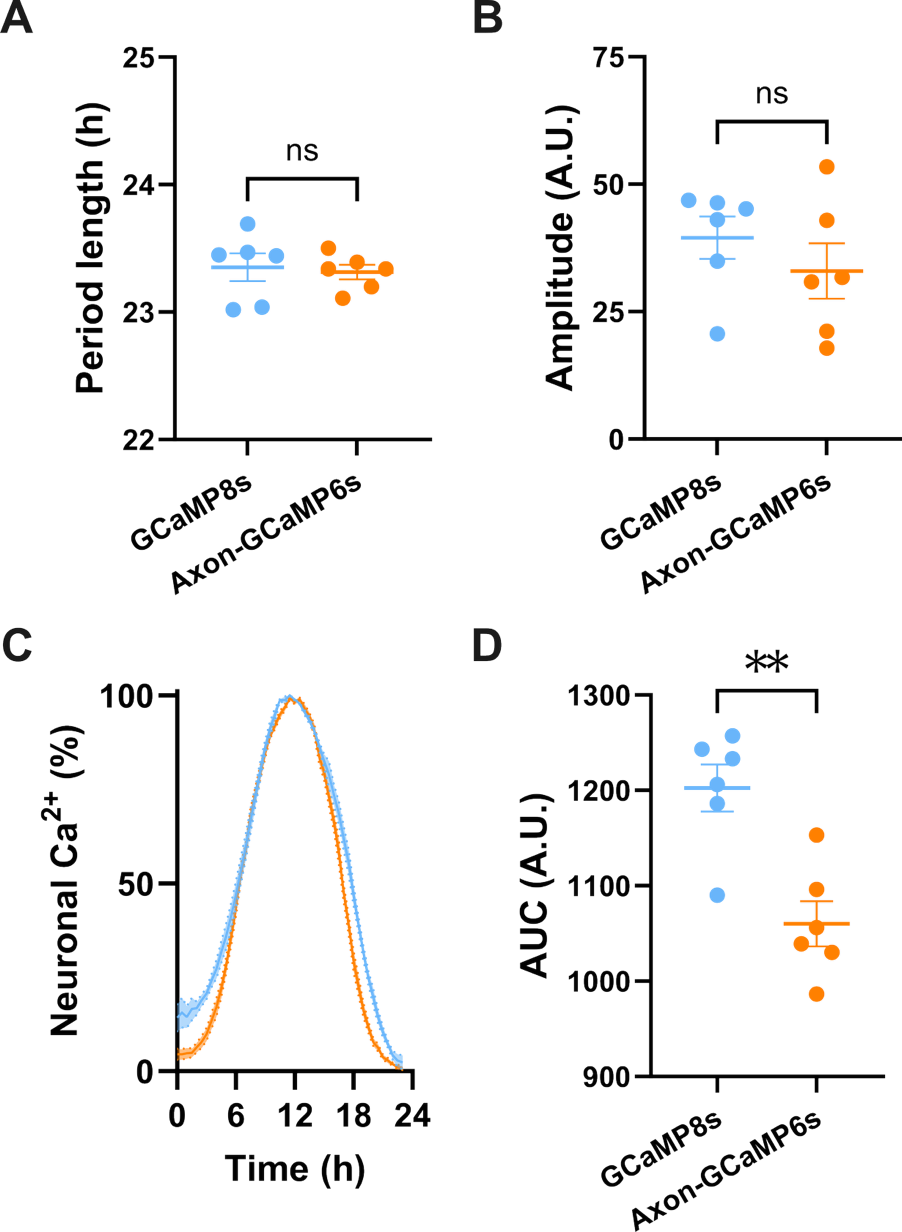


**Figure S7. Characterization of intracellular vs axonal Ca^2+^ reporters in SCN slices**

**(A)** Period length and **(B)** amplitude of traces represented in Fig 3B. **(C)** Profile of Syn-jGCaMP8s and Axonal-GCaMP6s signal, one cycle. Amplitude of traces represented in **Figure** **3B**. **(D)** Area under the curve of all traces used to produce the plot in panel c. In all panels n=6 per group, mean ±SEM; in panels **A**, **B**, **D** unpaired t-test; **=p<0.01, ns=p>0.05.

**
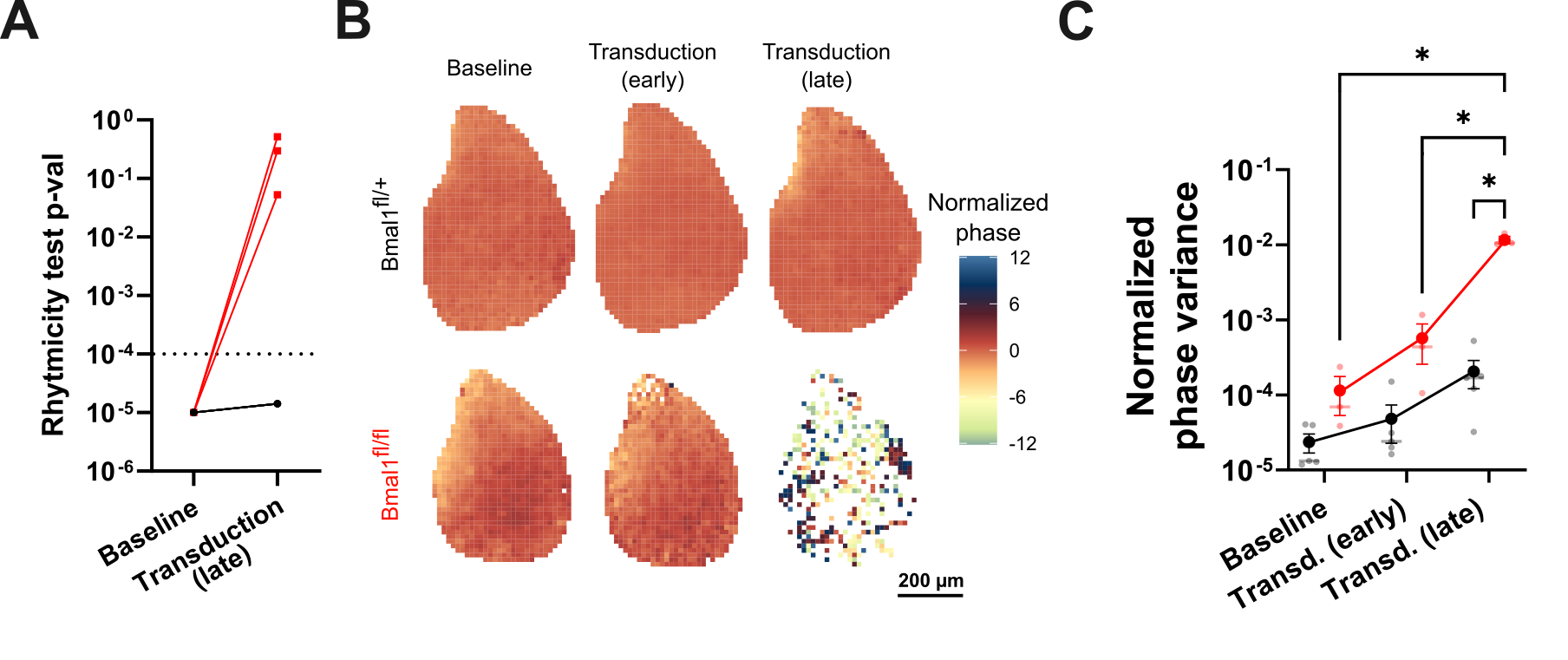
**

Figure S8. BMAL1 ablation impairs cellular rhythmicity in SCN slices

**(A)** Rhythmicity test (eJTK Biodare2) p-value of Axonal-GCaMP mean traces during baseline and late transduction phase. **(B)** Spatial map of normalized phase across genotype and reporter. **(C)** Normalized phase variance across genotype and experimental phases. Two-way ANOVA with Tukey multiple comparisons test. *=p<0.05, ***=p<0.001.


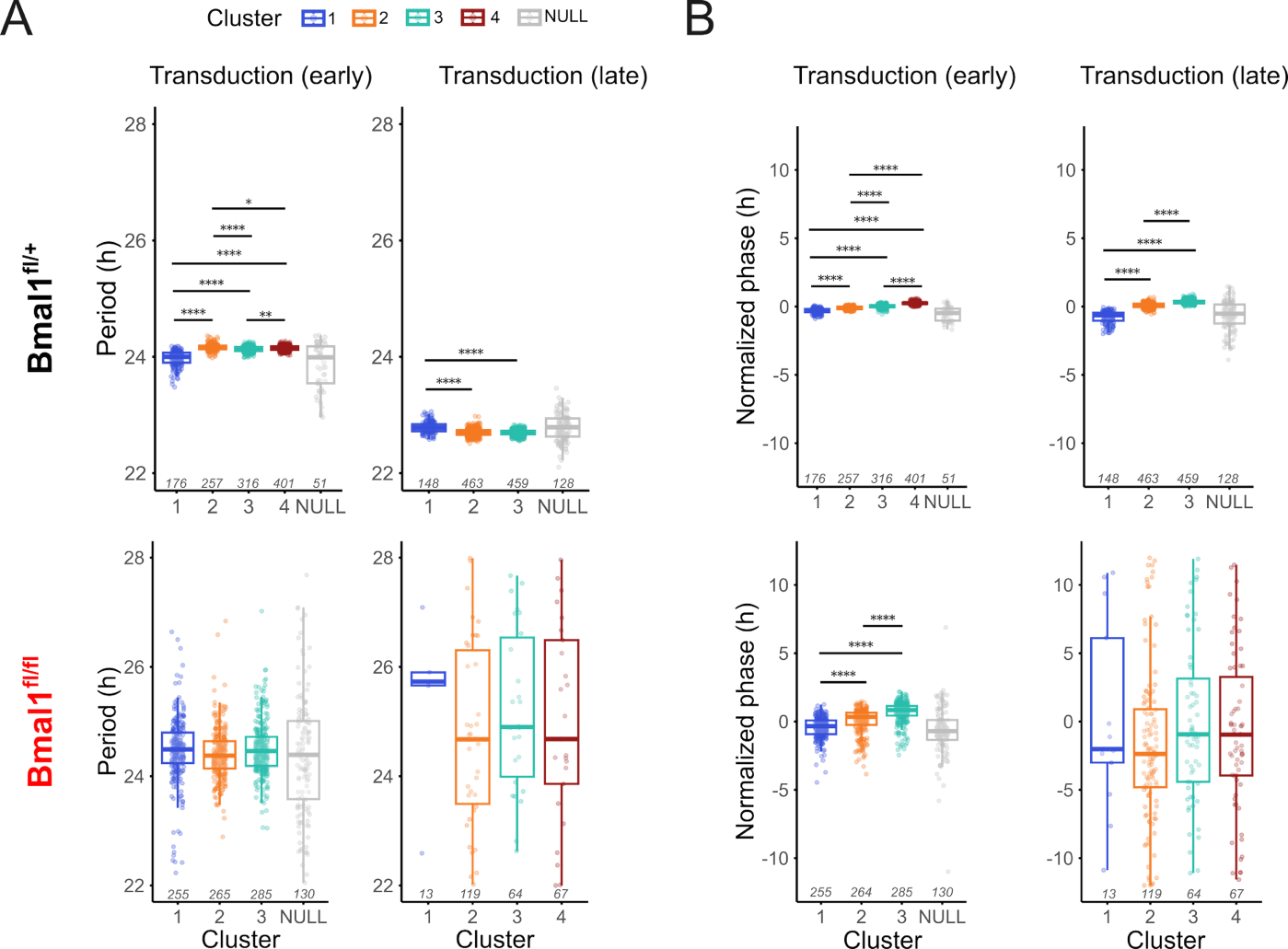


Figure S9. Comparison of period length and normalized phase across clusters

**(A)** Period length and **(B)** normalized phase values across different clusters in the early (left) and late (right) transduction phases. Each point represents the value from a single ROI. Number of ROIs for each cluster are indicated above the cluster label. Kruskal-Wallis test with Dunn’s multiple comparison; *=p<0.05, **=p<0.01, ****=p<0.01.

| **Package** | **Use** | **Source** |
| --- | --- | --- |
| astsa | Detrending functions | CRAN |
| base | Base R functions | R installation |
| behavr | timeseries analysis | CRAN |
| circular | Circular statistics | CRAN |
| cli | Progress bar | CRAN |
| config | Package development | CRAN |
| data.table | Data manipulation | CRAN |
| dplyr | Data manipulation | CRAN |
| DT | Data manipulation | CRAN |
| ggcorrplot | Correlation matrix | CRAN |
| ggetho | Plot layout | CRAN |
| ggplot2 | Plotting | CRAN |
| ggpubr | Plot layout | CRAN |
| golem | Package development | CRAN |
| grid | Report generation | R installation |
| igraph | Network analysis representation | CRAN |
| imputeTS | Timeseries interpolation | CRAN |
| knitr | Report generation | CRAN |
| minpack.lm | Non-linear least square calculation | CRAN |
| plyr | Data manipulation | CRAN |
| pracma | timeseries geometry | CRAN |
| pryr | read/write operations | https://github.com/hadley/pryr |
| purrr | Computation | CRAN |
| R6 | Pkg structure | CRAN |
| RImageJROI | Read ROI from ImageJ | CRAN |
| rlang | R processes | CRAN |
| rmarkdown | Report generation | CRAN |
| rstatix | Statistical test | CRAN |
| scales | Plotting layout | CRAN |
| sf | handle ROI | CRAN |
| shiny | Interactive environment | CRAN |
| shinydashboard | App layout | CRAN |
| shinydashboardPlus | App layout | CRAN |
| shinyjs | App Interaction | CRAN |
| shinyWidgets | App Interaction | CRAN |
| stats | Statistical operations | R installation |
| stringr | String manipulation | CRAN |
| svglite | SVG files generation | CRAN |
| tibble | Data manipulation | CRAN |
| tidygraph | Data manipulation | CRAN |
| tidyr | Data manipulation | CRAN |
| tools | File path manipulation | R installation |
| utils | Read/write operations | R installation |
| zeitgebr | Timeseries analysis | CRAN |
| zip | File output | CRAN |

**Table S1.** List of R packages used to develop the ClockCyteR and ClockCyteR.spatial packages. CRAN = Comprehensive R Archive Network.


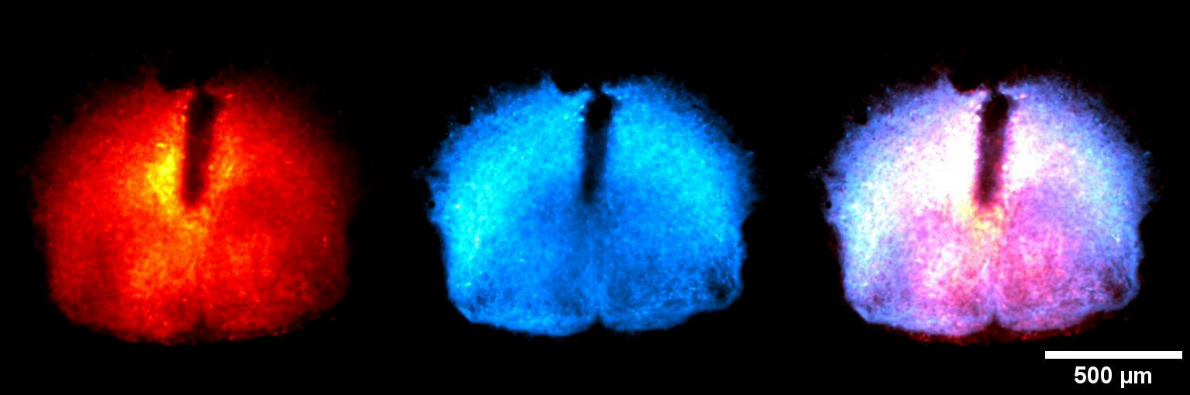
Movie S1. Multiplexed recording of neuronal and astrocytic reporters in an SCN slice

Live imaging recording of neuronal Ca^2+^ (Syn-jRCaMP1a; left, red), extracellular GABA (Syn-iGABASnFR; middle, cyan), and merge (right).


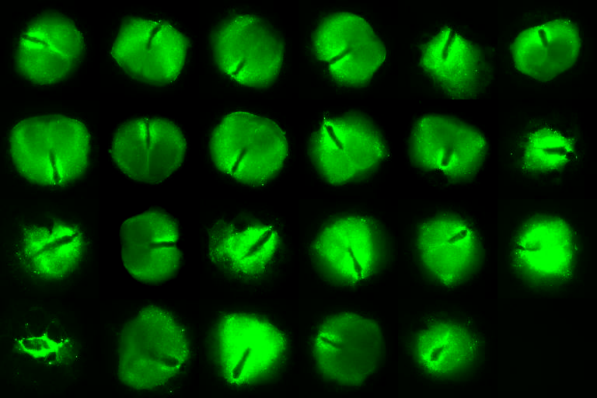


Movie S2. Live imaging of SCN neuronal Ca^2+^ from a 24-well plate

Live imaging recording of neuronal Ca^2+^ (Syn-jGCaMP8s) in SCN organotypic slices cultured in a 24-multiwell plate.


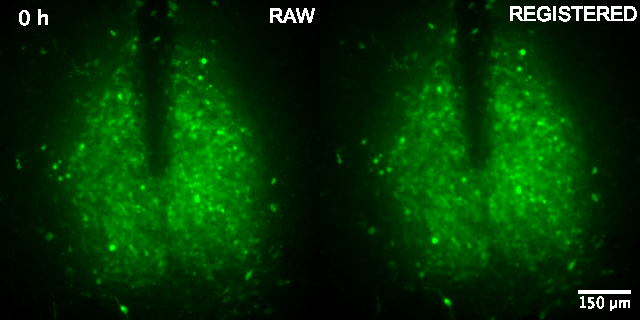


Movie S3. Comparison of timelapse data before and after motion registration

Live imaging recording of neuronal Ca^2+^ (Syn-jGCaMP8s) from SCN organotypic slice before and after running the description-based series registration 2d/3d+t plugin from FIJI.
